# Myoinhibitory peptide regulates feeding in the marine annelid *Platynereis*

**DOI:** 10.1186/s12983-014-0093-6

**Published:** 2015-01-07

**Authors:** Elizabeth A Williams, Markus Conzelmann, Gáspár Jékely

**Affiliations:** Max Planck Institute for Developmental Biology, Spemannstrasse 35, Tübingen, 72076 Germany

## Abstract

**Background:**

During larval settlement and metamorphosis, marine invertebrates undergo changes in habitat, morphology, behavior and physiology. This change between life-cycle stages is often associated with a change in diet or a transition between a non-feeding and a feeding form. How larvae regulate changes in feeding during this life-cycle transition is not well understood. Neuropeptides are known to regulate several aspects of feeding, such as food search, ingestion and digestion. The marine annelid *Platynereis dumerilii* has a complex life cycle with a pelagic non-feeding larval stage and a benthic feeding postlarval stage, linked by the process of settlement. The conserved neuropeptide myoinhibitory peptide (MIP) is a key regulator of larval settlement behavior in *Platynereis*. Whether MIP also regulates the initiation of feeding, another aspect of the pelagic-to-benthic transition in *Platynereis*, is currently unknown.

**Results:**

Here, we explore the contribution of MIP to the regulation of feeding behavior in settled *Platynereis* postlarvae. We find that in addition to expression in the brain, MIP is expressed in the gut of developing larvae in sensory neurons that densely innervate the hindgut, the foregut, and the midgut. Activating MIP signaling by synthetic neuropeptide addition causes increased gut peristalsis and more frequent pharynx extensions leading to increased food intake. Conversely, morpholino-mediated knockdown of MIP expression inhibits feeding. In the long-term, treatment of *Platynereis* postlarvae with synthetic MIP increases growth rate and results in earlier cephalic metamorphosis.

**Conclusions:**

Our results show that MIP activates ingestion and gut peristalsis in *Platynereis* postlarvae. MIP is expressed in enteroendocrine cells of the digestive system suggesting that following larval settlement, feeding may be initiated by a direct sensory-neurosecretory mechanism. This is similar to the mechanism by which MIP induces larval settlement. The pleiotropic roles of MIP may thus have evolved by redeploying the same signaling mechanism in different aspects of a life-cycle transition.

**Electronic supplementary material:**

The online version of this article (doi:10.1186/s12983-014-0093-6) contains supplementary material, which is available to authorized users.

## Background

Many organisms have a complex life cycle consisting of distinct stages that differ in form, physiology, behavior and habitat. Among benthic marine invertebrates, a common life cycle strategy, the biphasic life cycle, consists of a free-swimming larva that settles to the ocean floor and undergoes metamorphosis to a bottom-dwelling adult [1]. Marine invertebrate larval settlement is often coupled to the initiation of feeding or a change in diet [2]. These behavioral, physiological and morphological changes have to be tightly coordinated for the successful transition to a benthic life style. Knowledge of how this transition is regulated is important for understanding population structure in the ocean, life-history evolution, and how the environment influences animal life cycles [3-8].

The marine annelid *Platynereis dumerilii* has recently proven to be a useful marine invertebrate model for studying the molecular details of marine larval behavior, including settlement [9-12]. *Platynereis* has a biphasic life cycle with free-swimming, non-feeding larval (trochophore and nectochaete) stages and bottom-dwelling, feeding postlarval, juvenile and adult stages [13]. Larval settlement is followed by a period of growth and feeding, during which juvenile *Platynereis* add additional posterior segments. Cephalic metamorphosis, in which the first pair of parapodia are transformed into a second pair of tentacular cirri on the head, occurs after the juveniles have begun to add their sixth posterior segment [13-16].

Recently, we identified myoinhibitory peptide (MIP) as an inducer of rapid larval settlement behavior in *Platynereis* [11]. MIP is expressed in anterior chemosensory-neurosecretory neurons of the larva. Exogenous application of MIP inhibits the activity of the locomotor cilia, resulting in rapid sinking, and induces sustained contact with the substrate. *Platynereis* MIP belongs to an ancient neuropeptide family of Wamides, which are characterized by their amidated C-terminal tryptophan residue preceded by a small aliphatic residue [11,17]. Wamides are widespread among eumetazoans, except deuterostomes, and recently emerged as conserved regulators of life-cycle transitions [18]. For example, in some insects, MIP (also known as prothoracicostatic peptide (PTSP) or allatostatin-B (AST-B)) regulates ecdysone [19-21] and juvenile hormone levels [22], potentially influencing the timing of larval ecdysis and pupation. In cnidarians, including some corals and hydrozoans, GLWamide (also called metamorphosin) is known to induce larval settlement and metamorphosis [23-25].

How changes in feeding are regulated during marine life-cycle transitions is less well understood. Many neuropeptides are known to have roles in regulating different aspects of feeding [26-28]. MIPs/Wamides are also pleiotropic [29-35] and regulate aspects of feeding and gut muscle activity in some insects and cnidarians. The first MIP described had a myoinhibitory function on adult locust hindgut [36]. In several insects, MIP is expressed in the adult stage and can suppress muscle contractions of the hindgut [36-40]. MIP is also expressed in the stomatogastric nervous system of the adult crab, *Cancer borealis*, where it decreases the frequency of pyloric rhythm [41,42]. In addition, cnidarian GLWamide increases myoactivity in both hydra and sea anemone polyps, potentially influencing feeding [43,44]. Although none of these studies directly quantified feeding in the whole organism, MIP is a strong candidate for the regulation of feeding during marine life-cycle transitions.

Here, we study the expression and function of MIP in *Platynereis* during late larval (3-6 days) and early juvenile development (6-30 days). We found MIP expression in sensory neurons of the gut of 6 days and older *Platynereis*. We used both peptide-soaking and morpholino-mediated knockdown approaches to establish a role for MIP in the regulation of postlarval feeding and gut peristalsis. MIP treatment also resulted in faster juvenile growth, probably as a consequence of increased food ingestion and gut movement. Our results establish MIP as a pleiotropic neuropeptide in *Platynereis* that links behavioral and physiological components of a life-cycle transition.

## Results

### *Platynereis MIP* is expressed in the brain and gut of postlarvae, juveniles and adults

**Figure 1:**
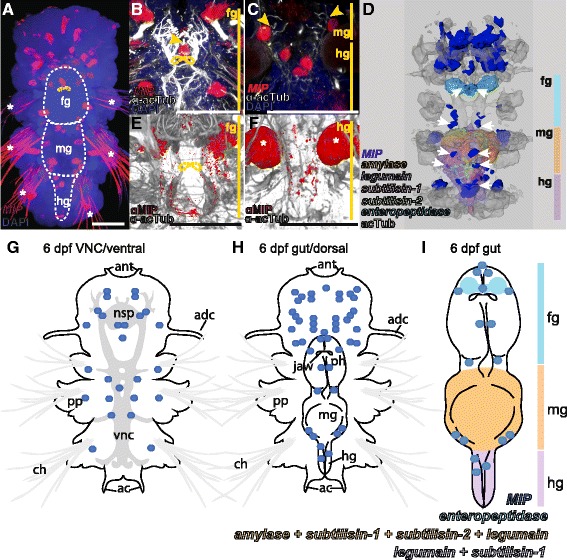
**MIP is expressed in the digestive system of 6 dpf** ***Platynereis*** **. (A)** Whole-mount RNA *in situ* hybridization (WMISH) for the *Platynereis MIP* precursor (red) counterstained with DAPI nuclear stain (blue). Ventral view of 6 dpf *Platynereis* with the image stacks corresponding to the ventral nerve cord (VNC) region not included in the maximal projection (outlined with white dashed line). White asterisks indicate background reflection from chaetae. **(B, C)** Close-up WMISH for the *Platynereis MIP* precursor (red) counterstained for acetylated tubulin (α-acTub) (white) and DAPI nuclear stain (blue). Yellow arrowheads indicate sensory dendrites of *MIP*-expressing cells. **(B)** Ventral view of the foregut. **(C)** Ventral view of the midgut and hindgut. **(D)** Dorsal view of surface representation of average *MIP* precursor expression domains registered to a 6 dpf nuclear stain reference template. Expression domains of *alpha-amylase, legumain protease precursor, subtilisin-1, subtilisin-2* and *enteropeptidase* are included as digestive system markers. White arrows indicate areas of *MIP* expression associated with the digestive system. **(E, F)** Immunostaining with *Platynereis* MIP antibody (red) counterstained with acetylated tubulin (grey) at 6 dpf, with the image stacks corresponding to the VNC region not included in the maximum projection to show only the digestive system. **(E)** Ventral view of foregut. **(F)** Ventral view of hindgut. White asterisks indicate background reflection from spinning glands. **(G-I)** Schematic representation of *MIP* precursor expression in 6 dpf *Platynereis*. **(G)** Ventral side. **(H)** Dorsal side. **(I)** *MIP* expression relative to expression of digestive system marker genes in the gut. In **(A, B, E)**, yellow dashed lines indicate the jaws. Scale bars: 50 μM. Abbreviations: fg, foregut; mg, midgut; hg, hindgut; ant, antenna; nsp, neurosecretory plexus; adc, anterior dorsal cirrus; pp, parapodia; vnc, ventral nerve cord; ch, chaetae; ac, anal cirrus; ph, pharynx.

Expression profiling of the *MIP* precursor gene by RNA *in situ* hybridization showed that *MIP* is expressed during both larval and postlarval development and continues to be expressed after cephalic metamorphosis, in the early adult stage (Figure 1A-C, G-H; Additional file 1). At 6 days and older, *MIP* is expressed in both the median brain and the trunk nervous system, in paired cells and also in single cells closer to the larval midline. We also found *MIP* expression in the digestive system, in the fore-, mid- and hindgut. The different regions of the gut are delineated by the differential expression patterns of *Platynereis* digestive system marker genes (Figure 1D, I; discussed below). The *MIP*-expressing cells in the gut have sensory dendrites that project toward the lumen of the gut (Figure 1B-C; Additional files 2, 3). In some of these dendrites we could even detect the *MIP* RNA *in situ* signal, allowing the unambiguous assignment of these acetylated tubulin-positive cellular projections to the MIP-expressing cells (Additional file 3).

In addition to *MIP*, we also attempted to characterize the expression of the *MIP receptor* in 6 dpf and older larvae. We previously described the expression of the MIP receptor in the head of 2 dpf *Platynereis* larvae [11], however, in older larvae and postlarvae, the levels of *MIP receptor* expression proved too low to detect reliably with our RNA *in situ* hybridization method. The low expression of the *Platynereis MIP receptor* is typical of most G protein-coupled receptor expression levels [45].

Immunostaining with an antibody against *Platynereis* MIP showed that in addition to the neurosecretory plexus of the brain, MIP peptide is transported throughout the ventral nerve cord. At 6 days post fertilization (dpf), MIP-expressing neurons in the digestive system innervate both the foregut and hindgut (Figure 1E, F). As larvae progress from 3 to 6 dpf, during which time the digestive system develops, MIP expression first emerges in the developing hindgut at 4 dpf, followed by the expression in the foregut at 6 dpf (Additional file 4A-L). By one month, MIP-expressing cells densely innervate the entire length of the gut, forming a nerve-net. Using an antibody against the conserved C-amidated dipeptide VWamide [11], we found similar immunolabeling in the brain, ventral nerve cord and gut of larvae of *Capitella teleta*, a distantly related annelid species [46] (Additional file 4M-P).

**Figure 2:**
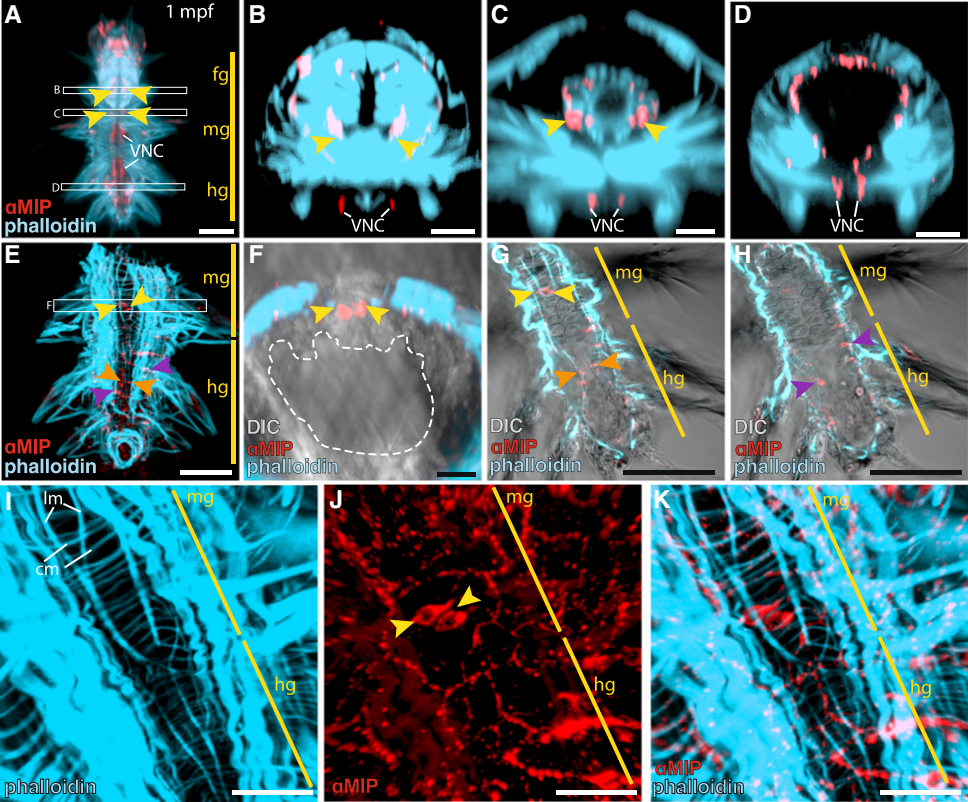
**Co-staining of MIP and phalloidin in 1 month post fertilization (mpf)** ***Platynereis.*** **(A-K)** Immunostaining of 1mpf *Platynereis* with an antibody raised against *Platynereis* MIP7 (red), counterstained with phalloidin. **(A)** Full body ventral view. White boxes indicate areas examined in cross-section in **(B-D)**. Yellow arrowheads in **(A-D)** indicate cell bodies of MIP-expressing neurons. **(B-D)** Apical views of 10 μM cross-sections of the foregut **(B)**, foregut-midgut boundary **(C)** and hindgut **(D). (E)** Ventral view of mid- and hindgut with ventral nerve cord region removed to expose digestive system. White box indicates area examined in cross-section in **(F)**. Yellow arrowheads in **(E-H, J)** indicate cell bodies of MIP-expressing neurons in the midgut. Orange and purple arrowheads in **(E, G, H)** mark cell bodies of MIP-expressing neurons in the hindgut. **(F)** Apical view of 10 μM cross-section in the midgut. Dashed white line marks the boundary between the gut lumen and epithelial cell layer. **(G, H)** Lateral layers of **(E)** indicating the MIP-expressing cell bodies of the midgut and hindgut that sit in the gut epithelium, just underlying the gut musculature. **(I-K)** Close-up ventral view of the mid- and hindgut. **(I)** Phalloidin staining shows the circular and longitudinal smooth muscle fibres of the gut. **(J)** MIP immunostaining. **(K)** Overlay of MIP immunostaining and phalloidin staining. The axons of MIP-expressing neurons run parallel to the muscle fibres of the gut. Scale bars in **(A, E, G-K)**: 50 μM, in **(B-D, F)**: 20 μM. Abbreviations: fg, foregut; mg, midgut; hg, hindgut; VNC, ventral nerve cord; DIC, differential interference contrast; cm, circular muscle fibre; lm, longitudinal muscle fibre.

By combining phalloidin staining and MIP immunostaining in *Platynereis* 1 month post fertilization (1 mpf), we could assess the location of MIP-expressing neurons in the gut in relation to the digestive system musculature. In the foregut and in the sphincter muscle that separates foregut from hindgut, MIP-expressing neurons are intermingled with the muscle tissue of the pharynx and sphincter (Figure 2A-C). In the mid- and hindgut, MIP-expressing neurons sit in the inner epithelial cell layer underlying the smooth muscles of the gut (Figure 2D-K). The axons of the MIP-expressing cells in the mid- and hind-gut of 1 month post fertilization (mpf) *Platynereis* run parallel to and just beneath the muscle fibers of both circular and longitudinal smooth muscles (Figure 2I-K). The spatial expression patterns of *Platynereis MIP* and MIP peptide suggest a potential role for MIP signaling in feeding and digestion during larval and early juvenile stages of the life cycle.

### Characterization of normal gut development and the initiation of feeding in postlarvae

At 6 dpf *Platynereis* postlarvae have a through gut with clearly recognizable fore-, mid- and hindgut regions (Figure 1). The foregut contains the muscular and extendable pharynx with the jaws and salivary glands (Additional file 2). Phyllodocid polychaetes, such as *Platynereis*, have an axial muscular pharynx consisting of circular, longitudinal and radial muscle fibers, which allow for complex sucking and swallowing movements [47,48]. The foregut-midgut boundary is marked by the presence of a sphincter muscle. This muscle showed regular contractions in larvae expressing a genetically encoded calcium indicator GCaMP6 (Additional file 5). The broad midgut does not show regionalization and is followed by a short and narrow hindgut.

To gain insight into the morphology and maturation of the *Platynereis* digestive system, we carried out whole-mount RNA *in situ* hybridization on 6 dpf, 14 dpf and 1 mpf *Platynereis* with marker genes selected from an ongoing broad RNA *in situ* hybridization screen, based on their expression in the digestive system at 6 dpf. The digestive system marker genes were identified through domain conservation, reciprocal BLAST and phylogenetic analyses as: extracellular digestive enzymes, peptidases *subtilisin-1 and subtilisin-2* (Peptidase_S8; Pfam domain: PF00082), the protease *enteropeptidase*, responsible for the activation of proteolytic enzymes [49], the polysaccharide-digesting enzyme *alpha*-*amylase*, and the intracellular digestive enzyme *legumain protease precursor* (Figure 1D, I; Additional files 6, 7, 8)*. Alpha-amylase* and *subtilisin-1* expression was restricted to the midgut at 6 dpf, but expanded to mid- and hindgut at 14 dpf and 1 mpf. *Legumain protease precursor* was constantly expressed in both mid- and hindgut from 6 dpf to 1 mpf, while *subtilisin-2* expression was restricted to the midgut from 6 dpf to 1 mpf. *Enteropeptidase* was the only gene with expression in the foregut, including the salivary glands, at 6 and 14 dpf. At 1 mpf, *enteropeptidase* remained strongly expressed in the foregut, but expression also extended to the mid- and hindgut. Registration of these marker gene expression patterns [50] at 6 dpf to a common nuclear stain reference scaffold, along with the average 6 dpf *MIP* expression, highlighted the close association of *MIP*-expressing cells with the digestive system at this stage (Figure 1D; Additional file 9).

We also looked at the change in expression of these digestive system marker genes and *MIP* across the *Platynereis* life cycle in stage-specific RNA-seq datasets [51]. With the exception of *legumain protease precursor*, the expression of all digestive system marker genes was undetectable in non-feeding larval stages but sharply increased between 4 and 10 dpf (Additional file 10). In accordance with a digestive function, these genes were strongly down-regulated in the adult non-feeding epitokes. *MIP* expression also increased sharply between 4 and 10 dpf, although it continued to be expressed in the non-feeding epitokes, suggesting further functional roles beyond feeding in *Platynereis*.

**Figure 3:**
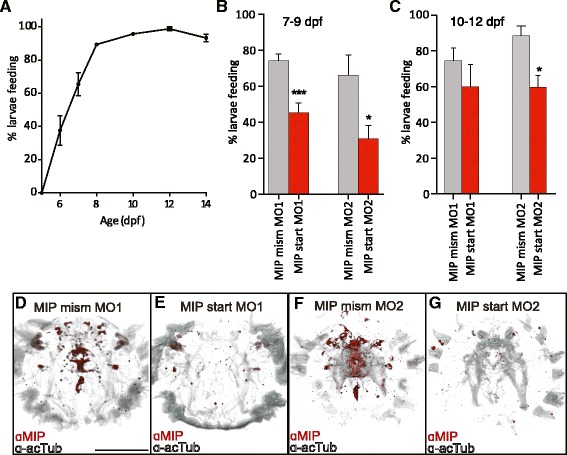
**Feeding in control and MIP knockdown larvae. (A)** Timecourse of initiation of feeding during *Platynereis* larval development. % larvae feeding on *Tetraselmis marina* algae between 5 – 14 dpf, n = 3 x 30 larvae. **(B)** % larvae feeding at 7 - 9 dpf following injection of MIP mismatch (n = 210 larvae mismatch MO1, 118 larvae mismatch MO2) or start (n = 424 larvae start MO1, 79 larvae start MO2) morpholinos **(C)** % larvae feeding at 10 - 12 dpf following injection of MIP mismatch (n = 115 larvae mismatch MO1, 72 larvae mismatch MO2) and start (n = 185 larvae start MO1, 62 larvae start MO2) morpholinos. Data in **(A-C)** are shown as mean +/- s.e.m. p-value cut-offs based on unpaired *t*-test: *** <0.001; ** < 0.01; * <0.05. **(D–G)** Anterior view of 6 dpf *Platynereis* injected with MIP mismatch or start morpholinos and immunostained with *Platynereis* MIP antibody (red) counterstained with acetylated tubulin (grey). Identical confocal microscopy and image processing parameters were applied to all images. Scale bar: 100 μm. Abbreviations: mism, mismatch; MO, morpholino; a-acTub, anti acetylated tubulin.

**Figure 4:**
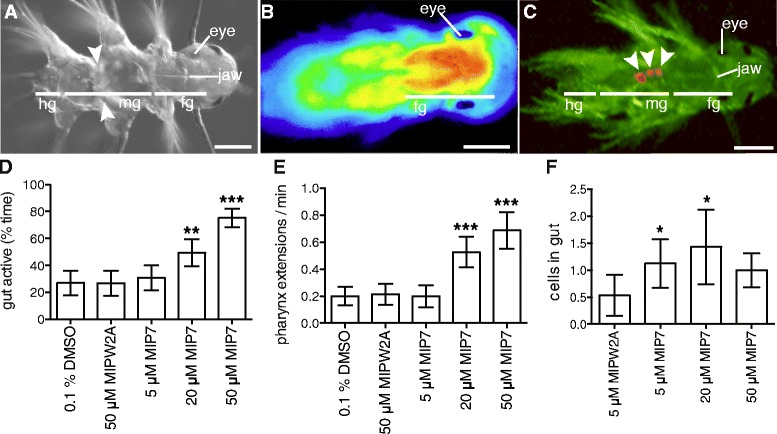
**Synthetic MIP treatment increases gut peristalsis, pharynx extension and ingestion in** ***Platynereis*** **. (A)** Differential interference contrast micrograph of 6.5 dpf *Platynereis*. White arrowheads indicate muscular contraction in the gut. **(B)** Calcium-imaging with GCaMP6 in 6.5 dpf *Platynereis* highlights muscular pharynx extension in the foregut. **(C)** Fluorescent micrograph of 7 dpf *Platynereis* with AF488 filter. White arrowheads indicate autofluorescent *Tetraselmis* cells in the gut. All images are dorsal views, with head to the right. **(D)** Gut activity as percentage of time in MIP-treated versus control 6.5 dpf *Platynereis*. **(E)** Number of pharynx extensions per minute in MIP-treated versus control 6.5 dpf *Platynereis*. **(F)** Number of *Tetraselmis marina* algae cells eaten per larvae in 30 min in MIP-treated versus control 7 dpf *Platynereis*. **(D-F)** Data are shown as mean +/- 95% confidence interval, n = 60 larvae. p-value cut-offs based on unpaired *t*-test: *** <0.001; ** < 0.01; * <0.05. MIPW2A is a control non-functional MIP peptide in which the two conserved tryptophan sites are substituted with alanines. Scale bars: 50 μm. Abbreviations: hg, hindgut; mg, midgut; fg, foregut.

Following settlement, *Platynereis* larvae have been reported to begin feeding between 5 – 8 dpf, with considerable variation between individuals [13,16]. Due to this variability, we decided to document feeding initiation in our own laboratory culture (Figure 3A). We added *Tetraselmis marina* microalgae to the larval cultures and documented feeding based on chlorophyll fluorescence in the gut (Figure 4C). Most larvae initiated feeding between 6 and 7 dpf; by 8 dpf, nearly all larvae had started feeding (Figure 3A).

### Knockdown of MIP delays the initiation of feeding in *Platynereis* larvae

To explore the function of MIP in the *Platynereis* digestive system, we employed morpholino microinjection to knockdown MIP expression. We used two different translation blocking morpholinos and two mismatch control morpholinos (Additional file 11). To test the effectiveness of MIP-knockdown, we immunostained knockdown and control larvae with an antibody against *Platynereis* MIP. We observed a strong reduction in MIP immunostaining in *Platynereis* MIP-knockdown larvae, but not in controls, up to at least 6 dpf (Figure 3D-G, Additional file 12). These experiments confirmed that the MIP morpholinos were capable of strongly reducing MIP expression.

Next, we documented feeding in MIP-knockdown and control larvae. Similar to untreated larvae, most larvae injected with a control morpholino had initiated feeding between 7-9 dpf, whereas a significantly lower number of MIP-knockdown larvae had food in the gut at 7-9 dpf. This effect was still observed between 10-12 dpf (Figure 3B-C).

To rule out that the reduced feeding in MIP-knockdown larvae is due to a developmental delay, we compared the morphology of the nervous system of MIP-knockdown and control larvae. There were no detectable differences in the nervous system of control larvae and MIP-knockdown larvae based on acetylated tubulin immunostainings at 6 dpf (Figure 3D-G; Additional file 13). We also treated uninjected larvae at different ages between 24 hours post fertilization (hpf) and 5 dpf with synthetic MIP peptide to see whether MIP-treated larvae initiate feeding sooner, indicating a potential developmental acceleration. MIP treatment did not significantly alter the timing of feeding initiation, even when food was available earlier than 5 dpf (Additional file 14). These experiments indicate a critical physiological role for MIP in the initiation of feeding behaviour in *Platynereis* postlarvae.

### MIP treatment has a myostimulatory effect on the digestive system of *Platynereis* postlarvae

In order to understand how the morpholino knockdown of MIP resulted in reduced larval feeding, we examined the effect of synthetic MIP treatment on postlarvae, focusing on the digestive system. Treatment of 6.5 dpf postlarvae with synthetic MIP caused a significant increase in gut peristalsis (Figure 4A, D; Additional file 15). MIP-treated postlarvae also displayed increased rates of pharynx extension (Figure 4B, E; Additional file 16).

To determine whether these effects on gut and pharynx movement resulted in increased ingestion of algal cells in MIP-treated postlarvae, we then scored the number of algal cells consumed by MIP-treated versus control 7 dpf postlarvae. Treatment with 5 μM and 20 μM MIP significantly increased postlarval algal cell consumption (Figure 4C, F; Additional file 17). Additionally, MIP-treated postlarvae have decreased locomotion, indicating a switch in the nervous system from a locomotory to a feeding program (Additional file 18). These results show that MIP up-regulates feeding activity and gut peristalsis.

### Long-term MIP treatment enhances growth in *Platynereis* postlarvae

**Figure 5:**
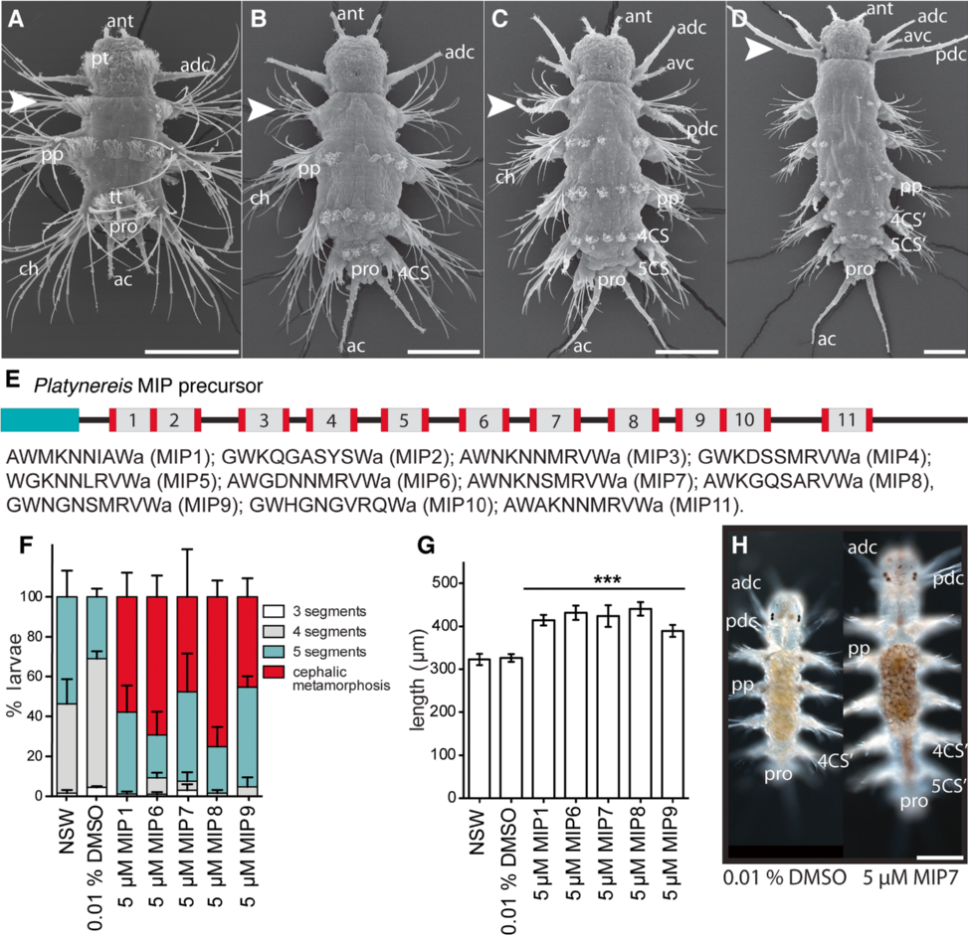
**Long-term treatment of** ***Platynereis*** **with synthetic MIP enhances growth and decreases time to cephalic metamorphosis. (A-D)** SEM images depicting posterior segment addition followed by cephalic metamorphosis in *Platynereis dumerilii*, dorsal views. White arrowhead indicates parapodia of the 1^st^ chaetigerous segment, which are transformed into the posterior tentacular cirri of the head during cephalic metamorphosis. **(A)** 6 dpf 3-segmented nectochaete larva. **(B)** 4-segmented errant juvenile. **(C)** 5-segmented errant juvenile. Cephalic metamorphosis has begun. **(D)** Cephalic metamorphosis is complete. **(E)** Schematic of the *Platynereis* MIP precursor protein. The N-terminal signal peptide (teal) and the predicted peptides (grey) flanked by basic cleavage sites (red) are shown. The predicted mature MIP peptide sequences are indicated below. **(F)** Percentage of 25 dpf *Platynereis* larvae/juveniles with 3, 4, 5 segments, or complete cephalic metamorphosis following exposure to 5 μM synthetic MIP peptides, from 4 dpf onwards. Data are shown as mean +/- s.e.m, n = 3 x 30 larvae. NSW, natural seawater control. **(G)** Total length of 25 dpf *Platynereis* larvae/juveniles exposed to 5 μM synthetic MIP peptides, from 4 dpf onwards. Data are shown as mean +/- 95% confidence interval, n = 90 larvae. **(F, G)** p-value cut-offs based on unpaired *t*-test: *** <0.001; ** < 0.01; * <0.05. **(H)** Differential interference contrast light micrographs of example control and MIP7-treated individuals at 1 month post fertilization. Scale bars: 100 μM. Abbreviations: ac, anal cirrus; adc, anterior dorsal cirrus; ant, antenna; avc, anterior ventral cirrus; ch, chaetae; pdc, posterior dorsal cirrus; pp, parapodia; pro, proctodeum; pt, prototroch; tt, telotroch; 4CS, 4^th^ chaetigerous segment; 5CS, 5^th^ chaetigerous segment; 4CS’, 4^th^ chaetigerous segment after cephalic metamorphosis; 5CS’, 5^th^ chaetigerous segment after cephalic metamorphosis.

Given the effect of MIP on the digestive system and feeding in *Platynereis* postlarvae, we next investigated the long-term effects of MIP treatment on postlarval growth. At approximately two weeks of age, feeding *Platynereis* begin to add new posterior segments [13]. After the development of the 5^th^ segment, juveniles undergo cephalic metamorphosis, a morphogenetic process in which the first chaetigerous segment loses its chaetae, develops a pair of tentacular cirri and fuses with the head (Figure 5A-D). The timing of cephalic metamorphosis and the addition of new segments vary between individuals. Even juveniles cultured individually showed variation in the timing of posterior segment addition, with segment number varying between 4 and 8 segments at 34 dpf (Additional file 19D). On a diet of *Tetraselmis*, the shortest interval for an individually-raised juvenile to develop an additional posterior segment was 4 days. The addition of new segments required that larvae begin to feed. Unfed larvae never develop beyond the 3-segmented stage (Additional file 19E). Growth in other nereid species depends on culture density [52-55]. We documented the growth of *Platynereis* juveniles cultured at different densities with excess food and determined the maximal density that still allowed optimal growth (3 larvae/ml) (Additional file 19 A-C). Under these conditions, juvenile *Platynereis* begin to develop the 5^th^ segment at 16 dpf, and start to undergo cephalic metamorphosis at 24 dpf. Morpholino knockdown methods are not applicable to such late stage animals, therefore we tested the effects of MIP treatment on errant juvenile growth. We found that the time to the addition of new posterior segments, and to cephalic metamorphosis, was reduced by sustained exposure to five different versions of mature MIP encoded by the *Platynereis MIP* preproneuropeptide gene (Figure 5E, F). At 25 dpf, some MIP-treated individuals had completed cephalic metamorphosis, while control individuals were yet to undergo cephalic metamorphosis. Comparing the body length of MIP-treated and control *Platynereis* at 25 dpf revealed that MIP-treated individuals were on average approximately 100 μM longer than control individuals (Figure 5G). The effect of MIP treatment on growth was only seen in the presence of food. In the absence of food, MIP treatment could not induce the addition of any new posterior segments, and postlarvae remained at the 3-segmented stage (Additional file 19E). Additionally, MIP-treated larvae, both fed and unfed, exhibited altered pigmentation of the gut and the body (Figure 5H, Additional file 19F, G).

## Discussion

Our results established the MIP neuropeptide as a regulator of postlarval feeding and gut activity in *Platynereis*. At 6 dpf, MIP is expressed in both the pharynx and the hindgut in neurons with a sensory morphology with dendrites projecting to the lumen. The MIP-expressing neurons of the *Platynereis* gut possess several hallmark features of mammalian enteroendocrine cells, including a scattered distribution, dendrites extending towards the gut lumen and long branching axons in the gut epithelial layer underlying the gut musculature [56]. These results are consistent with a model where MIP cells receive sensory signals from inside the mouth and the gut and respond by releasing MIP in a neurosecretory manner in the vicinity of the pharynx and hindgut muscles. However, given the use of bath-application and whole-body morpholino knock-down, we could not analyze the function of individual MIP-expressing cells. In principle, MIP-expressing neurons in other parts of the body may also affect gut activity by hormonal action.

Contrary to its name, MIP plays a myostimulatory role in the *Platynereis* digestive system*.* This may be the result of a direct effect whereby MIP directly acts on the digestive system musculature to increase the rate of pharynx extensions and peristaltic movements. Alternatively, the myostimulatory action of MIP may be caused indirectly through the regulation by MIP of other neurons in the gut, for example, in an as yet unidentified central pattern generator circuit responsible for regular gut contractions, as seen in crustaceans [57]. Knowledge of the spatial expression pattern of the MIP receptor in 6 dpf and older *Platynereis* larvae could help to resolve this.

Our results show that the increase in pharynx extensions in *Platynereis* postlarvae has a direct effect on the amount of food ingested. Increased gut peristalsis could promote the passage of food within the gut or the mixing of food with digestive enzymes, speeding up digestion. The fact that the highest concentration of MIP treatment, 50 μM, did not increase the amount of food ingested compared to control postlarvae is likely a result of the simultaneous reduction in locomotor activity caused by MIP treatment. At the highest concentrations of MIP (20 – 50 μM), increased gut peristalsis and pharynx extension activity may be offset by a decrease in locomotion, resulting in treated individuals encountering fewer algal cells.

The sustained expression of MIP in the gut and the long-term effects of MIP on juvenile growth indicate that MIP also has an important physiological role later in the life cycle. We interpret the enhancement of juvenile growth in long-term MIP treatment experiments to be a consequence of a sustained increase in feeding caused by MIP. Given its effect on both settlement and growth, MIP treatment may be a useful means of enhancing both larval settlement and juvenile growth in polychaete aquaculture [58].

Interestingly, MIP has a myostimulatory role in cnidarians and in the *Platynereis* digestive system, but a myoinhibitory role in the arthropod digestive system [36,41,44]. This could mean that either MIP was independently recruited to regulate gut activity in different phyletic lineages, or that the sign of the regulation switched during evolution. In the latter case, MIP would represent a conserved bilaterian gut peptide influencing feeding. Further comparative morphological and molecular studies of MIP cells and signaling pathways in a broader range of taxa will be needed to resolve this.

MIP regulates both settlement behavior and feeding, two aspects of the pelagic-to-benthic transition of the non-feeding *Platynereis* larvae. What could be the reason for the redeployment of the same peptidergic signal at different times during development and in different contexts? One possibility is that the anterior MIP-expressing sensory-neurosceretory cells of the larva and the MIP cells in the gut of the postlarva sense the same chemical cues released by potential food sources. Some marine larvae are induced to settle by their future juvenile food source [2]. Testing this hypothesis will require the identification of naturally occurring settlement cues and their corresponding receptors in *Platynereis*.

In *Platynereis*, juvenile feeding is an essential requirement for the completion of cephalic metamorphosis. In other polychaete species, where feeding often begins in the pelagic larval stage before settlement, feeding is also an essential component for settlement and metamorphosis. Starved larvae of *Capitella sp*., *Polydora ligia*, *Hydroides elegans* and *Phragmatopoma lapidosa* all lose or have decreased ability to complete settlement and metamorphosis [59-62]. Exploration of the roles of MIP in polychaete species with feeding larvae would increase our understanding of the links between MIP signaling, larval settlement and feeding.

## Conclusions

We have described a role for MIP in *Platynereis* postlarval feeding and established methods for studying the neuroendocrine regulation of feeding, providing the basis for future studies in this area. The amenability of *Platynereis* larvae to peptide treatments by soaking, their transparent body wall, and a neuropeptide complement that overlaps with that of both vertebrates and arthropods, make *Platynereis* an ideal model with which to study the neuroendocrine regulation of feeding in an evolutionary context.

## Methods

### *Platynereis* culture

*Platynereis* larvae were obtained from an in-house culture as previously described [15]. After fertilization of eggs, developing embryos and larvae were kept in an incubator at a constant temperature of 18°C with a regular light-dark cycle.

### *Platynereis* digestive system marker genes

Five genes with spatial expression domains restricted to the digestive system were identified from an ongoing RNA *in situ* hybridization screen of 48 hpf, 72 hpf and 6 dpf *Platynereis*. These genes were identified as *subtilisin-1* and *subtilisin-2* (Genbank Accession KM577672, KM577673), *alpha amylase* (Genbank Accession KM577675), *legumain-protease-precursor* (Genbank Accession KM77676) and *enteropeptidase* (Genbank Accession KM577674).

Genes were named according to their common conserved domains, reciprocal BLAST to the *Homo sapiens* peptidome, and neighbor-joining and maximum likelihood phylogenetic analyses (Additional files 7, 8). Genes were analyzed for the presence of a signal peptide assigned using the SignalP 4.1 server (http://www.cbs.dtu.dk/services/SignalP/) and conserved domains assigned by searches in the Pfam database with an e-value cutoff 1e-06 (http://pfam.xfam.org/search). To find additional sequences for use in phylogenetic analyses, the candidate gene sequences were used as queries in BLAST searches against the NCBI nr and Swissprot databases, taking the top 50 hits from each BLAST search. To diversify the range of phyla represented, BLAST searches were also performed with different restrictions, including ‘non-mammal’, ‘non-*Drosophila*’ and ‘Lophotrochozoans’. Sequence redundancy was reduced to 90% identity using CD-HIT [63]. Genes were aligned to candidate orthologues from other taxa with MUSCLE (http://www.ebi.ac.uk/Tools/msa/muscle/). Conserved regions of sequence alignment were select for phylogenetic analysis using Gblocks with minimal stringency settings [64]. Phylogenetic trees were constructed from trimmed sequence alignments using the neighbor-joining methods with 1000 bootstrap replicates in CLC Genomics Workbench 5.5.1 (CLC Bio, Qiagen), with a Gap Open Cost of 10 and a Gap Extension Cost of 1. Maximum likelihood trees with 100 bootstrap replicates were constructed using PhyML 3.0 using an LG substitution model and SPR and NNI tree searching methods [65]. Trees were inspected and taxa with long branches were removed to avoid long-branch attraction bias. The phylogenetic analyses were then re-run with the remaining taxa. We then went on to examine the expression of the digestive system marker genes in 6 dpf, 15 dpf and 1 mpf *Platynereis* by RNA *in situ* hybridization methods as described below.

### RNA *In situ* hybridization

Different developmental stages of *Platynereis* were collected for fixation for use in wholemount RNA *in situ* hybridization and immunostaining techniques. Individuals 6 days and older were relaxed using 1 M MgCl_2_ [66] prior to fixation. Postlarvae and juveniles that had begun feeding were starved for a few days prior to fixation to avoid the presence of autofluorescent algae cells in the gut, which interfere with fluorescent signals from immunostaining. All animals were fixed in 4% paraformaldehyde (PFA) in 0.1 M MOPS (pH 7.5), 2 mM MgSO_4_, 1 mM EGTA, 0.5 M NaCl for 1 h at room temperature. Fixed larvae were dehydrated through a MeOH series and stored in 100% MeOH at -20°C.

DIG-labelled antisense RNA probes for the *Platynereis MIP precursor* (JX513877), *MIP receptor* (JX513876), *alpha-amylase*, *subtilisin-1, subtilisin-2, legumain-protease precursor,* and *enteropeptidase* were synthesized from purified PCR products of clones sourced from a *Platynereis* cDNA library [51]. RNA *in situ* hybridization using nitroblue tetrazolium (NBT)/5-bromo-4-chloro-3-indolyl phosphate (BCIP) staining combined with mouse anti-acetylated-tubulin staining to highlight cilia and nervous system, followed by imaging with a Zeiss LSM 780 NLO confocal system and Zeiss ZEN2011 Grey software on an AxioObserver inverted microscope, was performed as previously described [50], with the following modification: fluorescence (instead of reflection) from the RNA *in situ* hybridization signal was detected using excitation at 633 nm in combination with a Long Pass 757 filter. Animals were imaged with a 40X oil objective.

### Image registration of RNA *in situ* hybridization patterns

We projected average *MIP* expression pattern of four 6 dpf individuals onto a common 6 dpf whole-body nuclear reference template generated from DAPI signal of 40 individuals as described previously for 72 hpf larvae [50]. Acetylated tubulin and expression patterns of digestive system marker genes of select individuals were also projected onto the reference template. Snapshots and video of the projected genes were generated in Blender 2.7.1 (http://www.blender.org/).

### Immunostaining

Immunohistochemistry with 1 μg/ml rabbit anti-MIP (AWNKNSMRVWamide) or cross-species anti-VWa, 0.5 μg/ml mouse anti-acetylated tubulin (Sigma) primary antibodies, and 1 μg/ml anti-rabbit Alexa Fluor® 647 (Invitrogen) and 0.5 μg/ml anti-mouse FITC (Jackson Immuno Research) secondary antibodies, was performed as previously described [67]. After the staining procedure, samples were mounted in 97% 2,2′-thiodiethanol (TDE).

For phalloidin stainings, we used freshly-PFA-fixed larvae dehydrated in 100% acetone for 5 minutes. Staining with rhodamine phalloidin (Molecular Probes) 1:100 in combination with the rabbit anti-MIP antibody was performed with the standard protocol adapted from [67]. After the staining procedure, samples were transferred to the mounting medium 87% glycerol containing 2.5 mg/mL of anti-photobleaching reagent DABCO (Sigma, St. Louis, MO, USA), as phalloidin-conjugated rhodamine destabilizes in TDE [68].

Confocal images were processed with Imaris 6.4 (Bitplane Inc., Saint Paul, USA) software. Raw image stacks and the *Platynereis* 6 dpf nuclear stained reference are available at the Dryad data repository.

### Calcium imaging

Fertilized eggs were injected with 500 ng/μl capped and polyA-tailed GCaMP6 [69] RNA generated from a vector (pUC57-T7-RPP2-GCaMP6) containing the GCaMP6 ORF fused to a 169 base pair 5′ UTR from the *Platynereis* 60S acidic ribosomal protein P2, as in [70]. Injection protocol is described in more detail in the ‘Morpholino Knockdown of *Platynereis* MIP’ section. Larvae were imaged with a 488 nm laser and transmission imaging with DIC optics on a Zeiss LSM 780 NLO confocal system on an AxioObserver inverted microscope (Additional file 5), or using a Zeiss AxioZoom V16 microscope with Hamamatsu Orca-Flash 4.0 digital camera (Additional file 13).

### RNA-Seq

RNA-Seq analysis of digestive enzyme and *MIP* precursor gene expression was performed on an existing dataset of 13 different stages spanning the *Platynereis* life cycle, from egg to mature adults. Methods used were described in [51].

### Documentation of normal feeding in *Platynereis*

To document variation in the commencement of feeding in *Platynereis* larvae from our laboratory culture, larvae were kept in Nunclon 6-well tissue culture dishes, with 10 ml sterile filtered seawater (FSW) per well. Each well contained 30 larvae. Larvae from 6 different batches, with different parents, were used in our analysis. Larvae were fed 5 μl *Tetraselmis marina* algae culture at 5 dpf. Larvae were then tested for feeding by checking for the presence of fluoresent *Tetraselmis* algae in the gut using a Zeiss Axioimager Z1 microscope with an AF488 fluorescent filter and a 20X objective. Larvae were checked for signs of feeding at 5.5, 6, 7, 8, 10, 12 and 14 dpf. After ingestion, algal cells can remain in the gut for up to 48 h before digestion causes a loss of fluorescence. Although larvae with a full gut can also be identified with normal light microscopy due to the transparent body wall, fluorescent microscopy enables the detection of even a single alga cell in the gut, due to the strong chlorophyll fluorescence of the *Tetraselmis* cells.

### Morpholino knockdown of *Platynereis* MIP

Two translation blocking morpholinos (MOs) and two corresponding 5 base pair mismatch control morpholinos were designed to target the *Platynereis-MIP-precursor* (GeneTools, LLC):

- Pdu-MIP-start MO1 TGATAGTGACGCGATC*CAT*TGGACT
- Pdu-MIP-mism MO1 TG*T*TAGTGAC*C*CG*T*TC*G*A*A*TGGACT
- Pdu-MIP-start MO2 CTAGTTCCTTCTCTCCCTCTTATCT
- Pdu-MIP-mism MO2 CTA*C*TT*G*CTT*G*TCTCC*G*T*G*TTATCT

Nucleotides altered in mismatch control morpholinos are in italics. Information on the position of the morpholinos in relation to the *MIP* start codon can be found in Additional file 11.

MOs were diluted in water with 12 μg/μl fluorescein dextran (*Mr* 10,000, Invitrogen) as a fluorescent tracer. 0.6 mM MOs were injected with an injection pressure of 600 hPa for 0.1 s and a compensation pressure of 35 hPa using Eppendorf Femtotip II needles with a Femtojet microinjector (Eppendorf) on a Zeiss Axiovert 40 CL inverted microscope equipped with a Luigs and Neumann micromanipulator. The temperature of developing zygotes was maintained at 16°C throughout injection using a Luigs and Neumann Badcontroller V cooling system and a Roth Cyclo 2 water pump.

For microinjection, fertilized *Platynereis* eggs developing at 16°C were rinsed 1 h after fertilization with sterile 0.2 μm filtered seawater (FSW) in a 100 μM sieve to remove the egg jelly, followed by a treatment with 70 μg/ml proteinase K for 1 min to soften the vitellin envelope. Following injection, embryos were raised in Nunclon 6-well plates in 10 ml FSW and their development was monitored daily.

Larvae were fed 5 μl *Tetraselmis marina* algal culture at 6 dpf. Feeding in 7 – 14 dpf injected larvae was assessed by checking for the presence of fluoresent *Tetraselmis marina* algae in the gut using a Zeiss Axioimager Z1 microscope with an AF488 fluorescent filter and a 20X objective. Larvae were checked for signs of feeding as described above every 24 h from 7 dpf on. We scored a minimum of 62 larvae (maximum 424 larvae) from a minimum of 3 separate microinjection sessions (with 3 different batches of larvae) for each translation-blocking and control morpholino. Photomicrographs of morpholino-injected larvae were also taken and larval body length was measured from these pictures using Image J 64 software. Some morpholino-injected larvae were also fixed at 6 days for immunostaining with the anti-MIP antibody (as described above) in order to assess morpholino specificity and effectiveness.

### Effect of synthetic MIP on *Platynereis* feeding behaviour

Peptide functions can be investigated in *Platynereis* larvae by bath application of synthetic neuropeptides [10,11]. To test whether synthetic MIP treatment increased developmental speed, leading to early initiation of feeding in *Platynereis* larvae, experiments were performed in Nunclon 6-well plates, with 10 ml FSW per well. Each control and peptide treatment was replicated across three wells, with 30 larvae per well. Larvae were treated with 5 μM MIP7 or controls at 24 hpf, 60 hpf, 4 dpf or 5 dpf, then fed at 4 or 5 dpf (depending on the age at which MIP treatment occurred) with 5 μl *Tetraselmis marina* algal culture. Larvae were fed at an earlier age due to the possibility of MIP treatment causing an earlier initiation of feeding. Larvae were checked for feeding by monitoring algal cell fluorescence in the gut as described above. Larvae were monitored from 5 or 5.5 dpf (depending on age at which larvae were first fed) until 7 or 8 dpf. A control non-functional MIP peptide (MIPW2A, AANKNSMRVAamide), in which the two conserved tryptophan sites were replaced with alanines (this prevents MIP from activating its receptor, see [11]) was also tested. A further control of larvae treated with DMSO alone was also included, as MIP peptides require DMSO to be dissolved in solution.

To test the effects of synthetic MIP peptide treatment on the digestive system of *Platynereis* larvae, we recorded videos of groups of 60 larvae at 6.5 dpf in a square glass cuvette 1.5 cm x 1.5 cm x 0.3 cm in 500 μl of FSW using a Zeiss AxioZoom .V16 microscope with Hamamatsu Orca-Flash 4.0 digital camera. For each treatment and control, 3 biological replicates (larval batches with different parentage, fertilized on different days) were carried out. We tested three concentrations of synthetic MIP: 5, 20 and 50 μM, plus 50 μM control non-functional MIP peptide MIPW2A and a 0.1 % DMSO control. A 2.5 min video at 10 frames per second was recorded 10 min after peptide or DMSO addition. Videos were analyzed manually in Fiji (Image J 1.48s, Wayne Rasband, http://imagej.nih.gov/ij). For each video, 20 larvae that remained within the frame of the video for the entire 2.5 min were scored for gut peristalsis and pharynx extension activity. Distance traveled and speed of the larvae was also measured using the MTrack2 plugin [71]. Significant differences in gut peristalsis, pharynx extension activity and locomotion in MIP-treated versus control larvae were tested in an unpaired *t* test.

To test the effects of synthetic MIP treatment on short-term ingestion of algal cells in *Platynereis* larvae, experiments were performed in Nunclon 24-well plates, with 2 ml FSW per well. Each control and peptide treatment was replicated across three wells, with 20 larvae per well. 7 dpf postlarvae were treated with 5 μM MIPW2A control peptide, 5 μM MIP, 20 μM MIP or 50 μM MIP for 10 min. Following this, 20 μl *Tetraselmis marina* algal culture was added to each well and larvae were left to feed for 30 min. All larvae were then immediately fixed in 0.5 mL 4% paraformaldehyde in 1X PBS with 0.01% Tween (PTw) for 1 hour. Following 4 washes in 1 ml PTw, larvae were mounted on glass slides and the number of algal cells in the digestive system of each larva was counted using a Zeiss Axioimager Z1 microscope with an AF488 fluorescent filter and a 20X objective. Significant differences in MIP-treated versus control larvae were tested in an unpaired *t* test.

### Scanning electron microscopy (SEM)

*Platynereis* larvae and juveniles of different developmental stages were fixed with 3% glutaraldehyde in 0.1 M phosphate buffer pH 7.2, rinsed in phosphate buffer, further fixed with 1 % osmium tetroxide in water and dehydrated in an ascending EtOH series over several days. Critical point drying with carbon dioxide was performed in a Polaron E 3000. The samples were coated with gold-palladium in a Balzers MED 010. Images were taken on a Hitachi S-800 Scanning electron microscope.

### Calculation of optimal culture density

The assessment of growth in larvae cultured individually was performed in a Nunclon 24-well tissue culture dish with 1 larva per well in 2 ml FSW. Larvae were fed from 6 dpf with 3 μl *Tetraselmis marina* algae culture. Larvae were scored under a dissection microscope for number of segments and cephalic metamorphosis every 48 h from 14 dpf to 34 dpf.

Documentation of growth in larvae cultured at different densities was carried out in Nunclon 6-well plates with 10 mL FSW/well and 30, 50 or 100 larvae per well. Three replicate wells were included for each culture density. Larvae were fed with surplus *Tetraselmis marina* algae throughout the experiment. Larvae were scored for segment number and cephalic metamorphosis every 4 days from 16 to 32 dpf.

### Long term treatment of *Platynereis* with synthetic MIP

To test the effect of synthetic MIP treatment on growth in *Platynereis* larvae, we again carried out experiments in Nunclon 6-well plates as described above, with 30 larvae per well and 3 replicate wells per treatment and control. 5 μM synthetic peptides were added at 4 dpf. Different versions of mature MIP peptide tested were: MIP1 – AWNKNNIAWamide, MIP6 – AWGDNNMRVWamide, MIP7 – AWNKNSMRVWamide, MIP8 – AWKGQSARVWamide, and MIP9 – GWNGNSMRVWamide. Larvae were also fed at 4 dpf with 5 μl *Tetraselmis sp.* algal culture. At 25 dpf (21 days after peptide addition), errant juveniles were scored for number of segments and cephalic metamorphosis (as above). Juveniles were also photodocumented using a Zeiss Axioimager Z1 microscope with differential interference contrast (DIC) and size of control and treated larvae (end of head to end of pygidium, excluding cirri) was measured in Fiji (Image J 1.48s, Wayne Rasband, http://imagej.nih.gov/ij).

We also tested the effects of synthetic MIP treatment on growth of unfed *Platynereis* larvae. Experiments were carried out as above, 5 μM MIP7 was added at 4 dpf. 5 μM MIPW2A and 0.1% DMSO were included as negative controls. At 24 dpf, errant juveniles were scored for number of segments and cephalic metamorphosis. Most unfed juveniles died between 24 and 28 dpf.

## Additional files

Additional file 1
**MIP expression in 14 dpf and 1 mpf** ***Platynereis.*** Whole-mount RNA *in situ* hybridization (WMISH) for the *Platynereis MIP* precursor (red) counterstained for acetylated tubulin (white) (A, C, D, E, G, H), or DAPI nuclear stain (blue) (B, F). All images shown in ventral view, with head to top. (A-D) 14 dpf, (E-H) 1 mpf. In (B-D) and (F-H), the ventral nerve cord region has been cut away to reveal digestive system. (C, G) close-up foregut (D, H) close-up mid- and hindgut. (I-K) Schematic of *MIP* precursor expression in 1 mpf *Platynereis*. (I) Ventral side. (J) Dorsal side. (K) *MIP* expression relative to expression of digestive system marker genes. In (B) and (F), white dashed lines indicate digestive system. In (B), (C), (F) and (G), yellow dashed lines mark jaws. Scale bars: 50 μm. Abbreviations: fg, foregut; mg, midgut; hg, hindgut; ant, antenna; nsp, neurosecretory plexus; adc, anterior dorsal cirrus; pp, parapodia; vnc, ventral nerve cord; ch, chaetae; ac, anal cirrus; ph, pharynx. Additional file 2
**Movie of MIP expression in foregut sensory cell of 6 dpf** ***Platynereis.*** Whole-mount RNA *in situ* hybridization (WMISH) for the *Platynereis MIP* precursor (red) counterstained for acetylated tubulin (white) and DAPI nuclear stain (blue). Ventral view of foregut. Yellow arrowhead marks sensory dendrite of MIP-expressing cell. Green arrows indicate salivary glands. Additional file 3
**Movie of MIP expression in mid- and hindgut sensory cells of 6 dpf** ***Platynereis.*** Whole-mount RNA *in situ* hybridization (WMISH) for the *Platynereis MIP* precursor (red) counterstained for acetylated tubulin (white) and DAPI nuclear stain (blue). Ventral view of mid- and hindgut. Yellow arrowheads mark sensory dendrites of MIP-expressing cells. Additional file 4
**MIP peptide expression timecourse in** ***Platynereis*** **, MIP peptide expression in** ***Capitella*** **larvae.** (A-L) Immunostaining of *Platynereis* larvae, postlarvae and juveniles with an antibody raised against *Platynereis* MIP7 (red), counterstained for acetylated tubulin (white) or DAPI nuclear stain (blue). All images ventral view with head to top. (A, B) 3 dpf, (C, D) 4 dpf, (E, F) 5 dpf, (G, H) 6 dpf, (I, J) 10 dpf, (K, L) 1 mpf. (M-P) Immunostaining of *Capitella teleta* Stage 9 larvae with an antibody raised against VWamide (red), counterstained for acetylated tubulin (white) or DAPI nuclear stain (blue). (M, N) ventral view, (O, P) lateral view. In (B, D, F, H, J, L, N), ventral nerve cord area has been removed to expose the underlying digestive system. Scale bars: 50 μm. White asterisks mark background fluorescence of parapodia, chaetae or spinning glands. White dashed lines indicate the developing digestive system. Abbreviations: fg, foregut; mg, midgut; hg, hindgut; pro, proctodeum; sto, stomodeum; dpf, days post fertilization; mpf, month post fertilization. Additional file 5
**Video of** ***Platynereis*** **6 dpf digestive system.** Calcium imaging with GCaMP6 shows muscular and neuronal activity in the digestive system of 6 dpf *Platynereis*. A sphincter muscle (indicated by yellow arrowheads) marks the boundary between foregut and midgut. Dorsal view with head to right. Additional file 6
***Platynereis*** **digestive system marker genes.** Schematic representation of *Platynereis* digestive enzyme genes *alpha*-*amylase, enteropeptidase*, *legumain protease precursor, subtilisin-1* and *subtilisin-2.* Signal peptide sequence and conserved domains are marked by coloured boxes, with e-value of an HMM search against the Pfam database (http://pfam.xfam.org) indicated in box. Length of amino acid sequence obtained is shown to the right of each gene. Additional file 7
**Phylogenetic trees of** ***Platynereis*** **digestive system marker genes.** Neighbour-joining trees with 1000 bootstrap repetitions (NJ-1000) and maximum likelihood trees with 100 bootstrap repetitions (ML-100) for digestive system markers *alpha amylase* (A, B), *enteropeptidase* (B, C), *legumain protease precursor* (D, E), and *subtilisin-1* and *subtilisin-2* (F, G). The positions of the *Platynereis* candidate genes are highlighted by green boxes. Bootstrap values are indicated at branch nodes. *Platynereis alpha amylase* and *legumain protease precursor* cluster with their counterparts in a fellow polychaete, *Capitella teleta*, within invertebrate-specific clades. The orthology of these genes was confirmed by reciprocal BLAST to the *Homo sapiens* peptidome. *Platynereis enteropeptidase* clusters in a weakly-supported group of annelid enteropeptidases, however the identity of this gene is confirmed by the presence of conserved MAM and trypsin domains (Additional file 6) [49]. *Platynereis subtilisin-*1 and *subtilisin-2* are intermingled with several bacterial, fungal, annelid and echinoderm sequences in a poorly resolved tree, suggesting a possible horizontal gene transfer event. *Subtilisin-1* and *–2* contain conserved peptidase domains (Additional file 6), including the presence of a catalytic triad, suggesting that they maintain enzymatic function in *Platynereis*. Additional file 8
**Expression timecourse of** ***Platynereis*** **digestive system marker genes.** Whole-mount RNA *in situ* hybridization (WMISH) for *Platynereis* digestive system marker genes (red) counterstained for DAPI nuclear stain (blue). Ventral view with the head to the top, ventral nerve cord region removed to show the digestive system. (A-C) *alpha amylase*, (D-F) *enteropeptidase*, (G-I) *legumain protease precursor*, (J-L) *subtilisin-2*, (M-O) *subtilisin-2*. (A, D, G, J, M) 6 dpf, (B, E, H, K, N) 14 dpf, (C, F, I, L, O) 1 mpf. White asterisks mark background fluorescence from jaws or chaetae. White dashed lines indicate the digestive system. Abbreviations: fg, foregut; mg, midgut; hg, hindgut. Scale bars: 50 μm. Additional file 9
**Movie of average MIP expression in 6 dpf** ***Platynereis*** **in relation to digestive system marker genes.** Surface representation of the average expression domains of MIP (blue) at 6 dpf relative to the axonal scaffold (grey). Surface representations of digestive enzyme genes are added sequentially to mark the digestive system. In order of appearance, genes are: *alpha amylase* (orange), *enteropeptidase* (cyan), *subtilisin-2* (green), *legumain protease precursor* (purple), and *subtilisin-1* (pink). Movie starts in dorsal view with head to the top and rotates around anterior-posterior axis. Additional file 10
**Expression of** ***MIP*** **and digestive system marker genes throughout** ***Platynereis*** **life cycle.** Histograms of normalized gene expression generated from RNA-Seq libraries of 13 different life-cycle stages, from egg to sexually mature epitokes. (A) *alpha amylase* (B) *enteropeptidase*, (C) *legumain protease precursor*, (D) *subtilisin*-*1* (E) *subtilisin-2* and (F) *MIP*. Life-cycle stages during which *Platynereis* feeds are 10 dpf, 15 dpf, 1 mpf pre-cephalic metamorphosis, 1 mpf post-cephalic metamorphosis, and 3 mpf atokous adult. Habitat transitions are also indicated under the histograms: pelagic, free-swimming; benthic, crawling on bottom; pelagobenthic, may switch between swimming and crawling. Additional file 11
**Target sites of** ***Platynereis*** **MIP start morpholinos.** The sequence of the 5′ region of the *Platynereis MIP precursor* gene is shown. Binding sites of MIP start morpholinos (MOs) 1 and 2 are outlined in red. The start codon for the MIP precursor peptide is underlined and in bold. The 5′ predicted peptide sequence is indicated in grey. MIP start MO1 binds to the start codon region of the gene, while MIP start MO2 binds in the upstream 5′-UTR region. Additional file 12
**MIP knockdown in** ***Platynereis*** **is confirmed by anti-MIP immunostaining.** Immunostaining of 6 dpf *Platynereis* with an antibody raised against *Platynereis* MIP7 (red). All images in apical view. (A-C) Larvae injected with mismatch control morpholino 1. (D-K) Larvae injected with MIP start morpholino 1. (L-N) Larvae injected with mismatch control morpholino 2. (O-Q) Larvae injected with MIP start morpholino 2. Injection of morpholinos targeting the start site of MIP results in reduced expression of MIP peptide compared to injection of control morpholinos. Black asterisks in (A-K) indicate background fluorescence caused by the oxidation of eye pigment proteins in larvae exposed to strong light. Scale bars: 20 μM. Additional file 13
**MIP knockdown** ***Platynereis*** **are morphologically similar to control** ***Platynereis.*** (A,B) Ventral view of 6 dpf *Platynereis* injected with MIP mismatch morpholino 1 (A) or MIP start morpholino 1 (B) and immunostained with *Platynereis* MIP antibody (red) counterstained with acetylated tubulin (grey). Identical confocal microscopy and image processing parameters were applied to all images. Scale bar: 100 μm. Abbreviations: mism, mismatch; MO, morpholino; α-acTub, anti acetylated tubulin. Additional file 14
**Early treatment with MIP does not induce early onset feeding.** Graphs of % larvae with food in gut over time after treatment with 5 μM MIP, 5 μM control MIPW2A or 0.1% DMSO from (A) 24 hpf, (B) 60 hpf, (C) 4 dpf, or (D) 5 dpf. Data are shown as mean +/- s.e.m., n = 3 x 30 larvae. p-value cut-offs based on unpaired *t*-tests indicated no significant difference in initiation of feeding between MIP-treated and control larvae. MIPW2A is a control non-functional MIP peptide in which the two conserved tryptophan sites are substituted with alanines. Additional file 15
**Movie of gut peristalsis in 6.5 dpf** ***Platynereis.*** Dorsal view with head to the right. Extension of pharynx can also be seen at 10 sec and 15 sec. Additional file 16
**Movie of pharynx extension in 6.5 dpf** ***Platynereis.*** Dorsal view with head to the right. Calcium imaging with GCaMP6 shows muscular extension of the pharynx in the foregut. Top panel is differential interference contrast (DIC), bottom panel is colour-coded with Jet-LUT colour map in Image J, most intense signal in red. Additional file 17
**Movie of feeding in 7 dpf** ***Platynereis.*** *Platynereis* (green) feeds on autofluorescent *Tetraselmis* algae (red). Movie was filmed on a Zeiss AxioZoom microscope with AF488 fluorescent filter. Additional file 18
**MIP treatment decreases distance traveled and speed of 6 dpf** ***Platynereis.*** (A) Distance travelled by MIP-treated versus control 6.5 dpf *Platynereis*. (B) Speed of MIP-treated versus control 6.5 dpf *Platynereis*. Data are shown as mean +/- 95% confidence interval, n = 60 larvae. p-value cut-offs based on unpaired *t*-test: *** <0.001; ** < 0.01; * <0.05. MIPW2A = control non-functional MIP peptide in which the two conserved tryptophan sites are substituted with alanines. Additional file 19
**Long-term growth of** ***Platynereis.*** Addition of posterior segments in errant juveniles raised at a density of (A) 3 larvae/mL (30 larvae in 10mL NSW), n = 3 x 30, (B) 5 larvae/mL (50 larvae in 10 mL NSW), n = 3 x 50, or (C) 10 larvae/mL (100 larvae in 10 mL NSW), n = 3 x 100. (A-C) Data are shown as mean + s.e.m. All larvae were fed from 6 dpf onwards. ‘Cephalic metamorphosis’ encompasses all worms that have completed cephalic metamorphosis and have 5 or more chaetigerous segments. (D) Addition of posterior segments in errant juveniles raised individually. 24 larvae were raised individually in a 24-well tissue culture dish with 2 mL NSW per well. Note: Larva #13 died prior to 12 dpf. (E) No addition of posterior segments in unfed 5 μM MIP7-treated and control errant juveniles. Data shown are mean + s.e.m, n = 3 x 30 larvae. NSW, 0.22 μM filtered natural seawater. MIPW2A is a control non-functional MIP peptide in which the two conserved tryptophan sites are substituted with alanines. (F, G) Differential interference contrast (DIC) light micrographs of example unfed (F) control and (G) MIP-treated individuals at 24 dpf. Scale bar: 50 μm.

## Abbreviations

4CS: 4th chaetigerous segment
4CS’: 4th chaetigerous segment after cephalic metamorphosis
5CS: 5th chaetigerous segment
5CS’: 5th chaetigerous segment after cephalic metamorphosis
α-acTub: anti acetylated tubulin
ac: anal cirrus
adc: anterior dorsal cirrus
ant: antenna
AST-B: allatostatin-B
avc: anterior ventral cirrus
ch: chaetae
cm: circular muscle fibre
DAPI: 4′,6-Diamidino-2-Phenylindole, Dihydrochloride
DIC: differential interference contrast
DMSO: dimethyl sulfoxide
dpf: days post fertilization
fg: foregut
FSW: 0.2 μm filtered seawater
hg: hindgut
hpf: hours post fertilization
lm: longitudinal muscle fibre
mg: midgut
MIP: myoinhibitory peptide
mism: mismatch
MO: morpholino
mpf: months post fertilization
nsp: neurosecretory plexus
pdc: posterior dorsal cirrus
ph: pharynx
pp: parapodia
pro: proctodeum
pt: prototroch
PTSP: prothoracicostatic peptide
PTw: 1X PBS with 0.01% Tween
SEM: scanning electron microscopy
sto: stomodeum TDE, 2,2′-thiodiethanol
tt: telotroch
vnc: ventral nerve cord
