# Supplementary material for: Myoinhibitory peptide regulates feeding in the marine annelid Platynereis
Source: Front Zool. 2015 Jan 7;12:1. doi: 10.1186/s12983-014-0093-6 (PMC4307165; doi:10.1186/s12983-014-0093-6)
Supplement: Additional file 1 — MIP expression in 14 dpf and 1 mpf Platynereis. Whole-mount RNA in situ hybridization (WMISH) for the Platynereis MIP precursor (red) counterstained for acetylated tubulin (white) (A, C, D, E, G, H), or DAPI nuclear stain (blue) (B, F). All images shown in ventral view, with head to top. (A-D) 14 dpf, (E-H) 1 mpf. In (B-D) and (F-H), the ventral nerve cord region has been cut away to reveal digestive system. (C, G) close-up foregut (D, H) close-up mid- and hindgut. (I-K) Schematic of MIP precursor expression in 1 mpf Platynereis. (I) Ventral side. (J) Dorsal side. (K) MIP expression relative to expression of digestive system marker genes. In (B) and (F), white dashed lines indicate digestive system. In (B), (C), (F) and (G), yellow dashed lines mark jaws. Scale bars: 50 μm. Abbreviations: fg, foregut; mg, midgut; hg, hindgut; ant, antenna; nsp, neurosecretory plexus; adc, anterior dorsal cirrus; pp, parapodia; vnc, ventral nerve cord; ch, chaetae; ac, anal cirrus; ph, pharynx. [file 12983_2014_93_MOESM1_ESM.pdf]

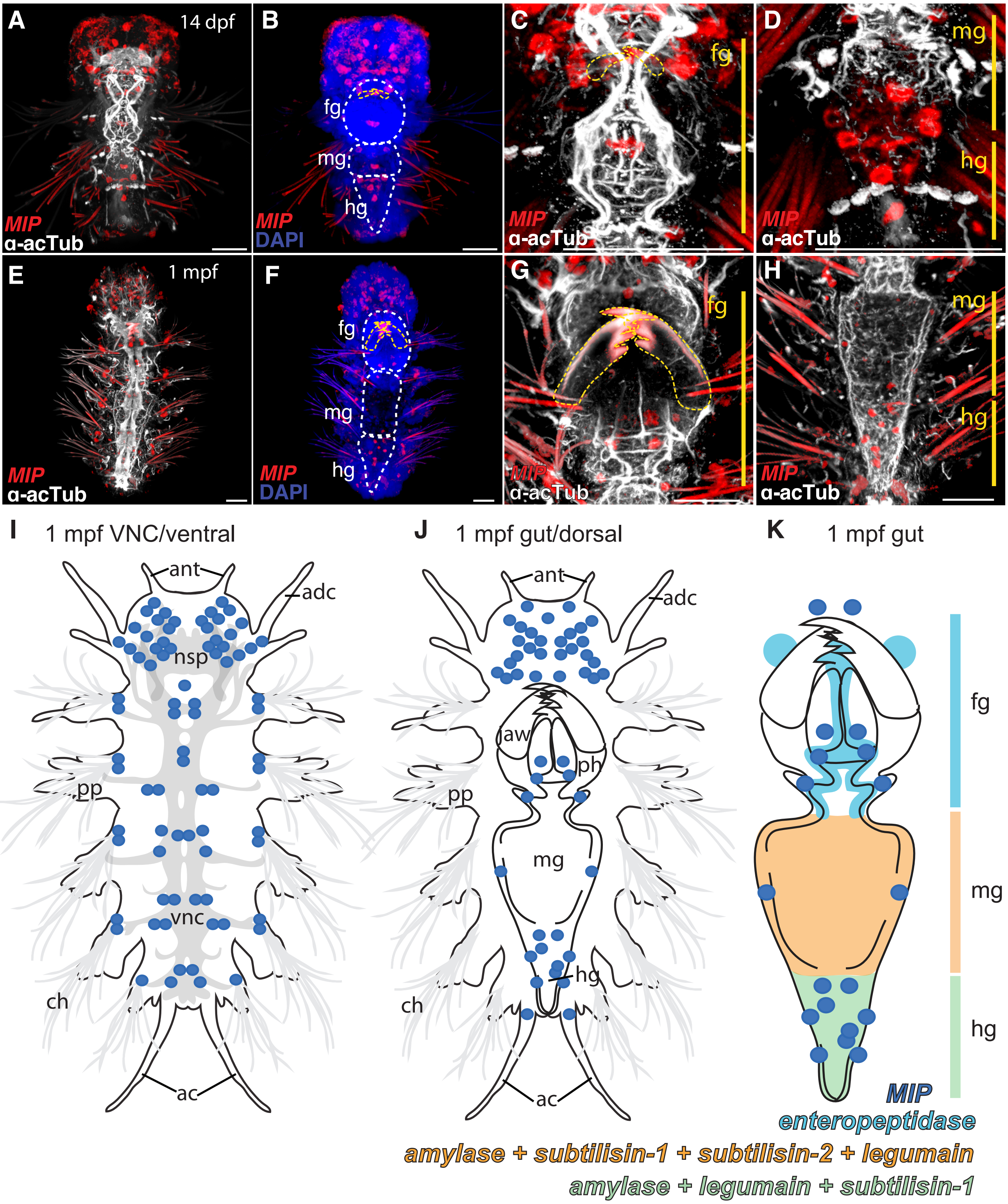

A
14 dpf
MIP
α-acTub
B
fg
mg
hg
MIP
DAPI
C
fg
MIP
α-acTub
D
mg
hg
MIP
α-acTub
E
1 mpf
MIP
α-acTub
F
fg
mg
hg
MIP
DAPI
G
fg
MIP
α-acTub
H
mg
hg
MIP
α-acTub
I
1 mpf VNC/ventral
ant
adc
nsp
pp
vnc
ch
ac
J
1 mpf gut/dorsal
ant
adc
jaw
ph
pp
mg
hg
ch
ac
K
1 mpf gut
fg
mg
hg
MIP
enteropeptidase
amylase + subtilisin-1 + subtilisin-2 + legumain
amylase + legumain + subtilisin-1
