# Supplementary figures and images for: Myoinhibitory peptide regulates feeding in the marine annelid Platynereis
Source: Front Zool. 2015 Jan 7;12:1. doi: 10.1186/s12983-014-0093-6 (PMC4307165; doi:10.1186/s12983-014-0093-6)

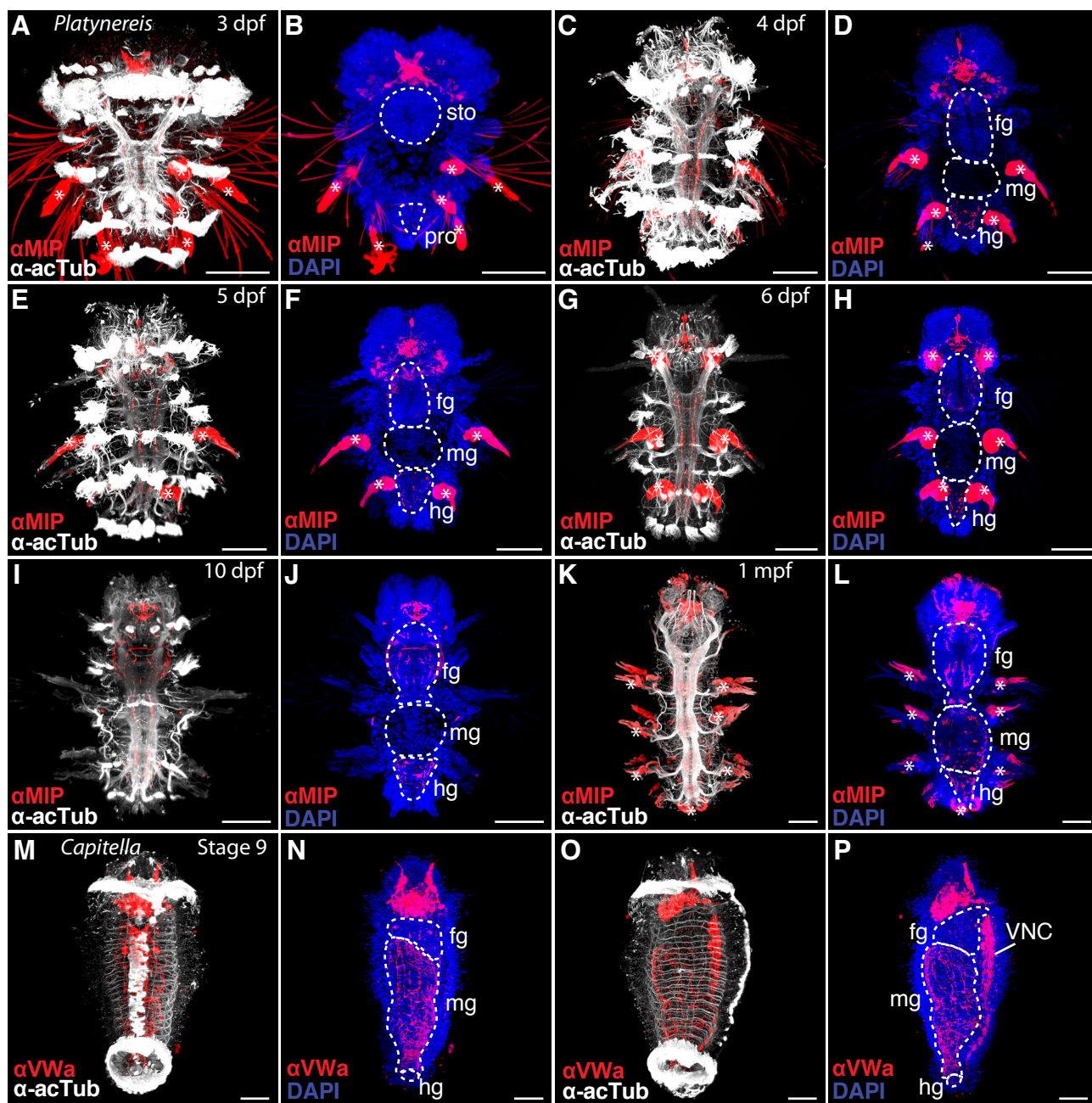

Supplement: Additional file 4 — MIP peptide expression timecourse in Platynereis , MIP peptide expression in Capitella larvae. (A-L) Immunostaining of Platynereis larvae, postlarvae and juveniles with an antibody raised against Platynereis MIP7 (red), counterstained for acetylated tubulin (white) or DAPI nuclear stain (blue). All images ventral view with head to top. (A, B) 3 dpf, (C, D) 4 dpf, (E, F) 5 dpf, (G, H) 6 dpf, (I, J) 10 dpf, (K, L) 1 mpf. (M-P) Immunostaining of Capitella teleta Stage 9 larvae with an antibody raised against VWamide (red), counterstained for acetylated tubulin (white) or DAPI nuclear stain (blue). (M, N) ventral view, (O, P) lateral view. In (B, D, F, H, J, L, N), ventral nerve cord area has been removed to expose the underlying digestive system. Scale bars: 50 μm. White asterisks mark background fluorescence of parapodia, chaetae or spinning glands. White dashed lines indicate the developing digestive system. Abbreviations: fg, foregut; mg, midgut; hg, hindgut; pro, proctodeum; sto, stomodeum; dpf, days post fertilization; mpf, month post fertilization. [file 12983_2014_93_MOESM4_ESM.pdf]

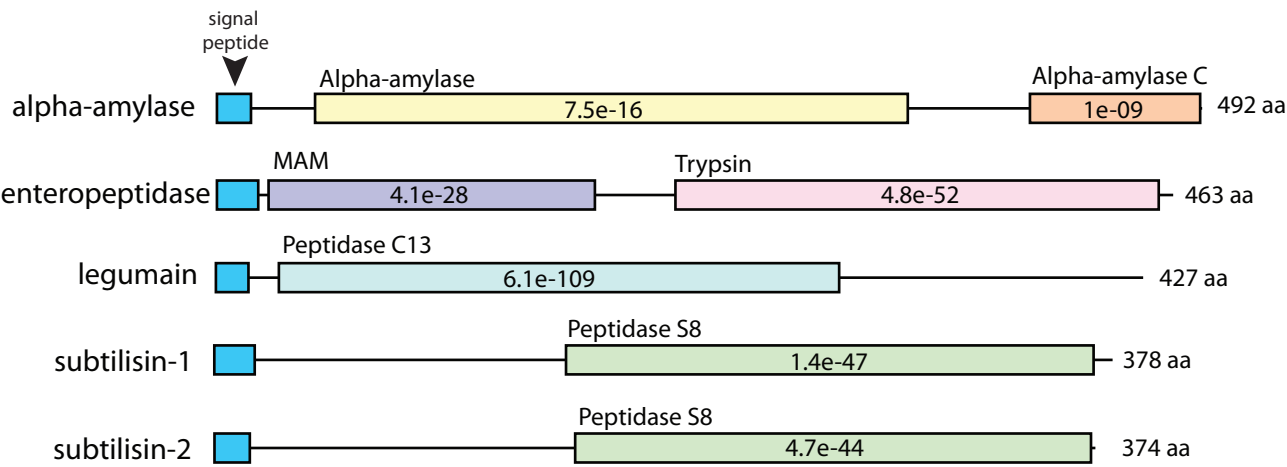

Supplement: Additional file 6 — Platynereis digestive system marker genes. Schematic representation of Platynereis digestive enzyme genes alpha-amylase, enteropeptidase, legumain protease precursor, subtilisin-1 and subtilisin-2. Signal peptide sequence and conserved domains are marked by coloured boxes, with e-value of an HMM search against the Pfam database (http://pfam.xfam.org) indicated in box. Length of amino acid sequence obtained is shown to the right of each gene. [file 12983_2014_93_MOESM6_ESM.pdf]

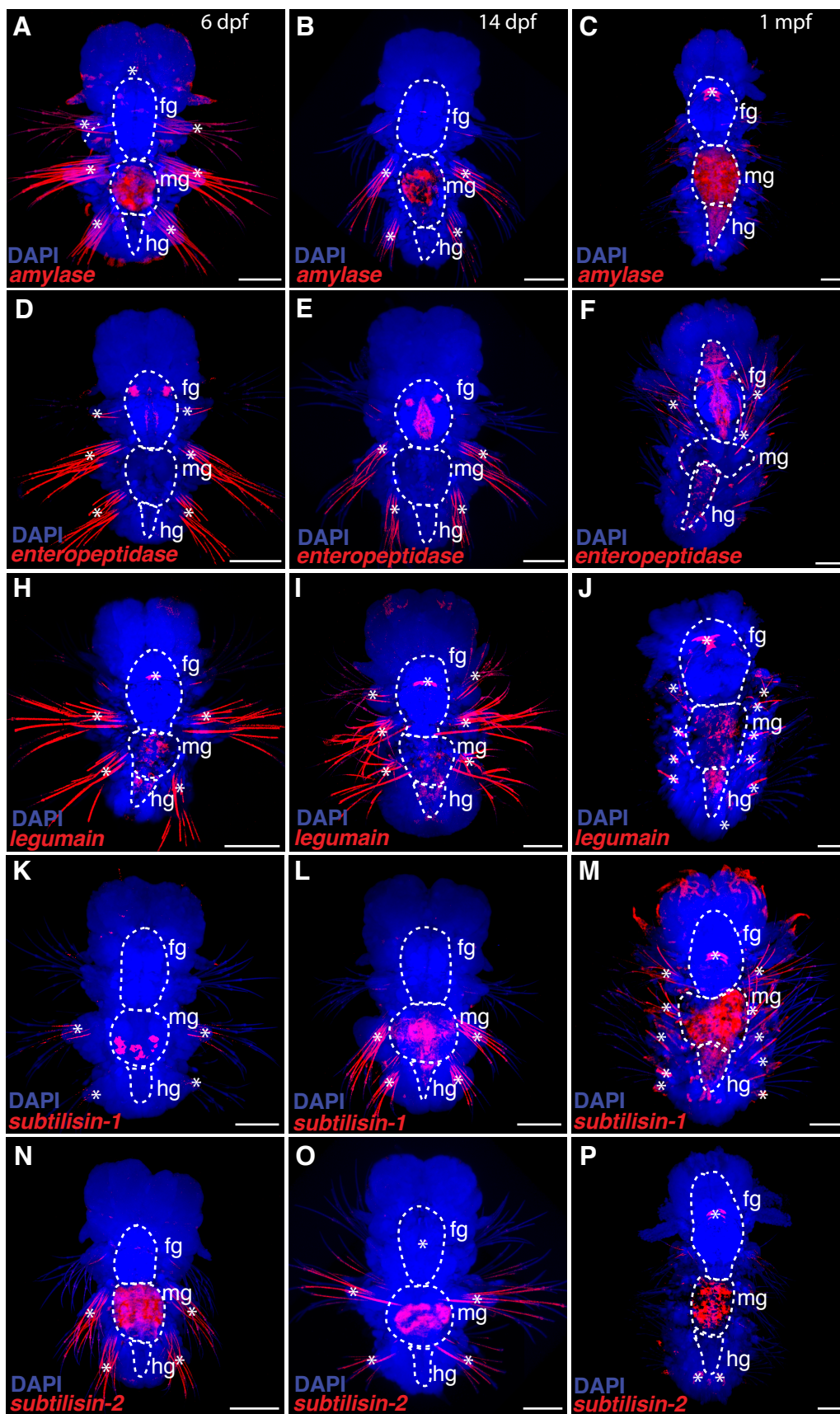

Supplement: Additional file 8 — Expression timecourse of Platynereis digestive system marker genes. Whole-mount RNA in situ hybridization (WMISH) for Platynereis digestive system marker genes (red) counterstained for DAPI nuclear stain (blue). Ventral view with the head to the top, ventral nerve cord region removed to show the digestive system. (A-C) alpha amylase, (D-F) enteropeptidase, (G-I) legumain protease precursor, (J-L) subtilisin-2, (M-O) subtilisin-2. (A, D, G, J, M) 6 dpf, (B, E, H, K, N) 14 dpf, (C, F, I, L, O) 1 mpf. White asterisks mark background fluorescence from jaws or chaetae. White dashed lines indicate the digestive system. Abbreviations: fg, foregut; mg, midgut; hg, hindgut. Scale bars: 50 μm. [file 12983_2014_93_MOESM8_ESM.pdf]

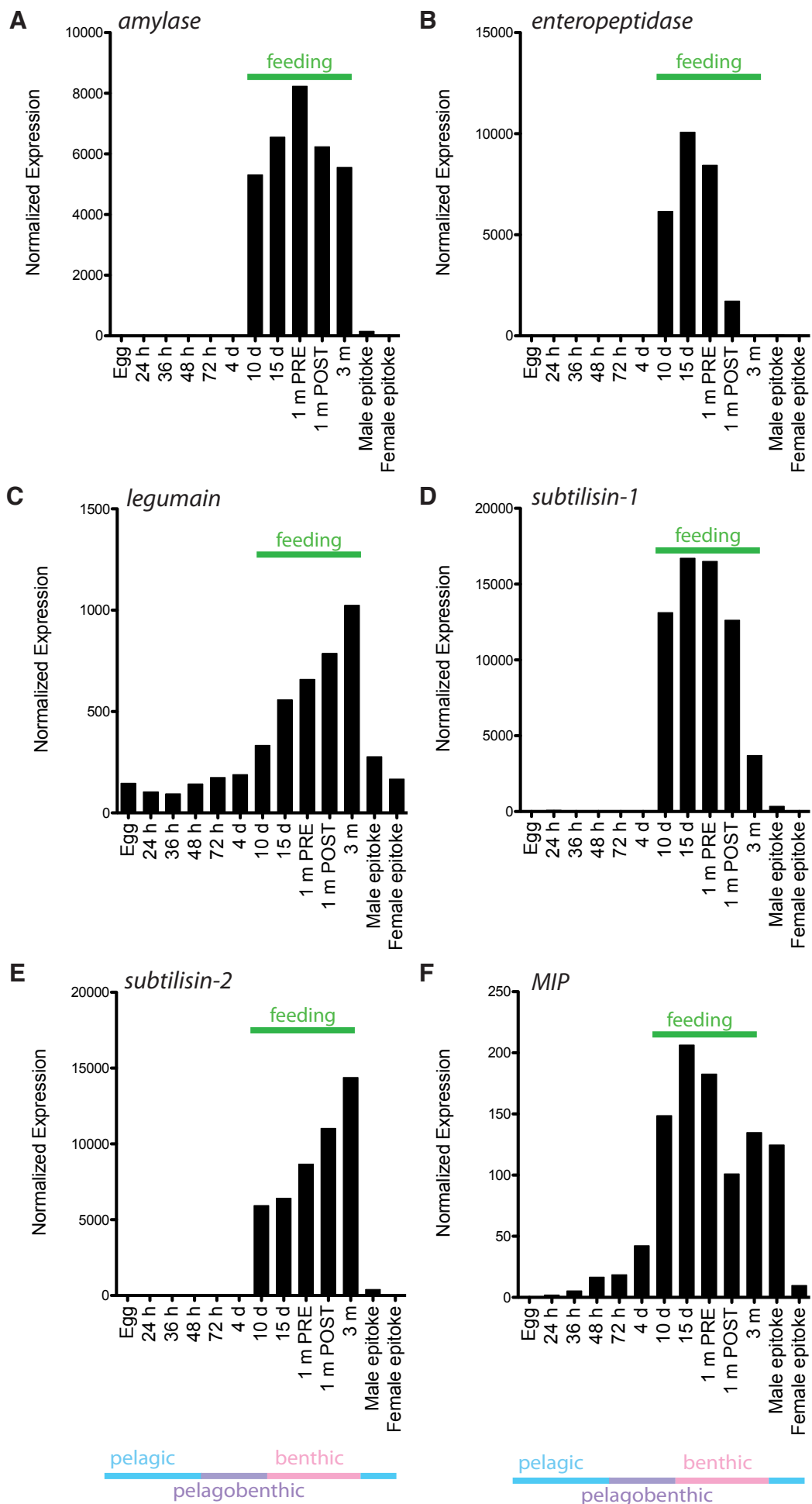

Supplement: Additional file 10 — Expression of MIP and digestive system marker genes throughout Platynereis life cycle. Histograms of normalized gene expression generated from RNA-Seq libraries of 13 different life-cycle stages, from egg to sexually mature epitokes. (A) alpha amylase (B) enteropeptidase, (C) legumain protease precursor, (D) subtilisin-1 (E) subtilisin-2 and (F) MIP. Life-cycle stages during which Platynereis feeds are 10 dpf, 15 dpf, 1 mpf pre-cephalic metamorphosis, 1 mpf post-cephalic metamorphosis, and 3 mpf atokous adult. Habitat transitions are also indicated under the histograms: pelagic, free-swimming; benthic, crawling on bottom; pelagobenthic, may switch between swimming and crawling. [file 12983_2014_93_MOESM10_ESM.pdf]

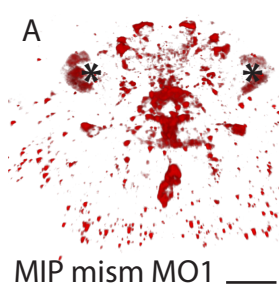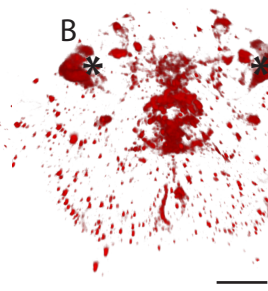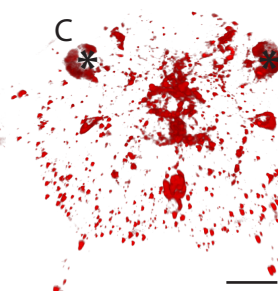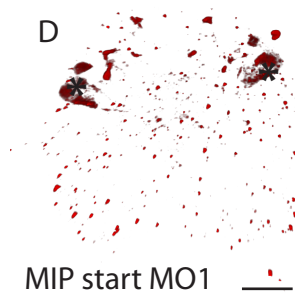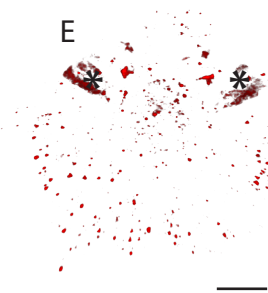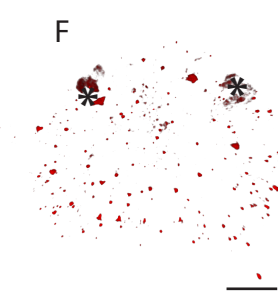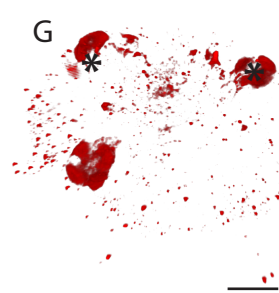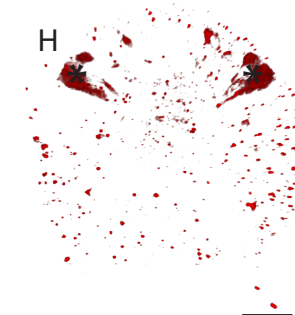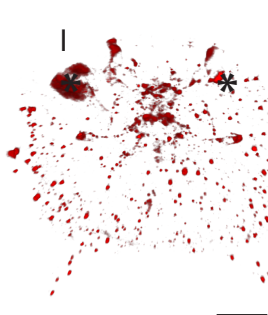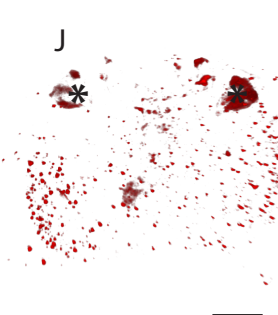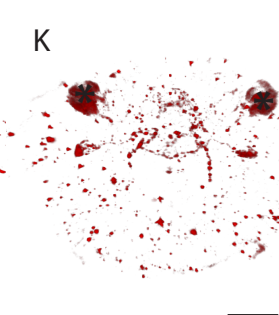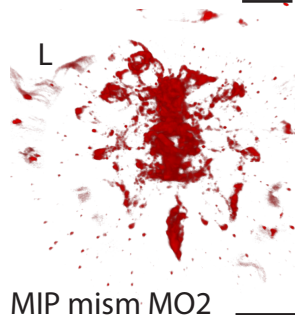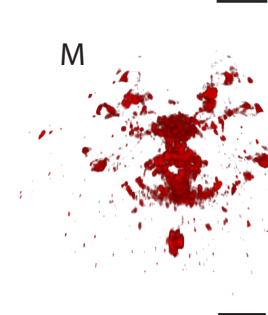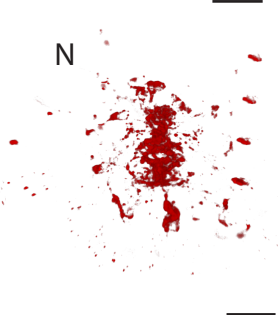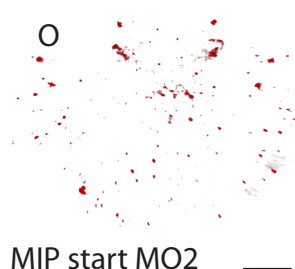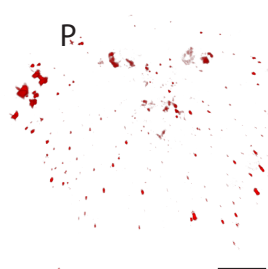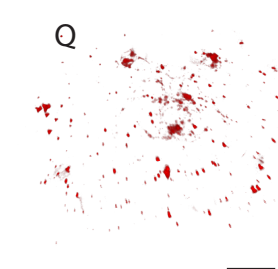

Supplement: Additional file 12 — MIP knockdown in Platynereis is confirmed by anti-MIP immunostaining. Immunostaining of 6 dpf Platynereis with an antibody raised against Platynereis MIP7 (red). All images in apical view. (A-C) Larvae injected with mismatch control morpholino 1. (D-K) Larvae injected with MIP start morpholino 1. (L-N) Larvae injected with mismatch control morpholino 2. (O-Q) Larvae injected with MIP start morpholino 2. Injection of morpholinos targeting the start site of MIP results in reduced expression of MIP peptide compared to injection of control morpholinos. Black asterisks in (A-K) indicate background fluorescence caused by the oxidation of eye pigment proteins in larvae exposed to strong light. Scale bars: 20 μM. [file 12983_2014_93_MOESM12_ESM.pdf]

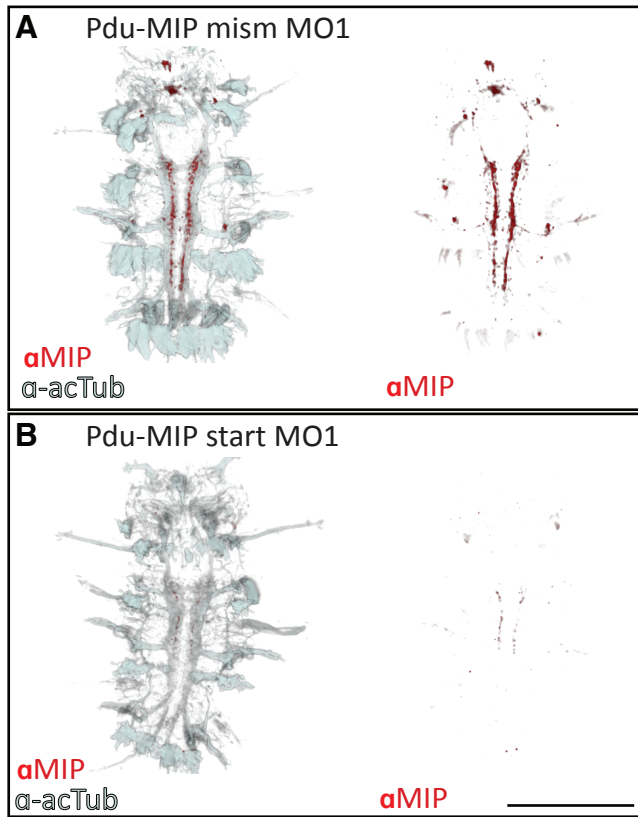

Supplement: Additional file 13 — MIP knockdown Platynereis are morphologically similar to control Platynereis. (A,B) Ventral view of 6 dpf Platynereis injected with MIP mismatch morpholino 1 (A) or MIP start morpholino 1 (B) and immunostained with Platynereis MIP antibody (red) counterstained with acetylated tubulin (grey). Identical confocal microscopy and image processing parameters were applied to all images. Scale bar: 100 μm. Abbreviations: mism, mismatch; MO, morpholino; α-acTub, anti acetylated tubulin. [file 12983_2014_93_MOESM13_ESM.pdf]

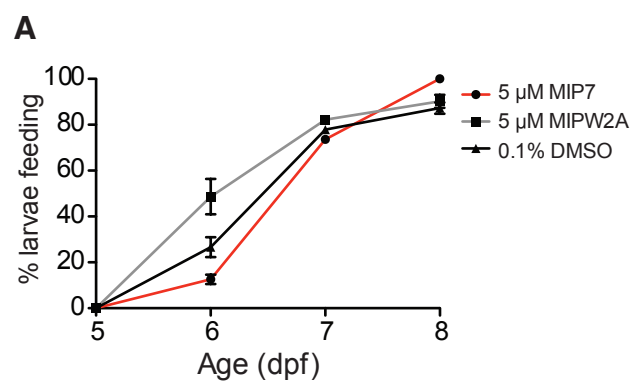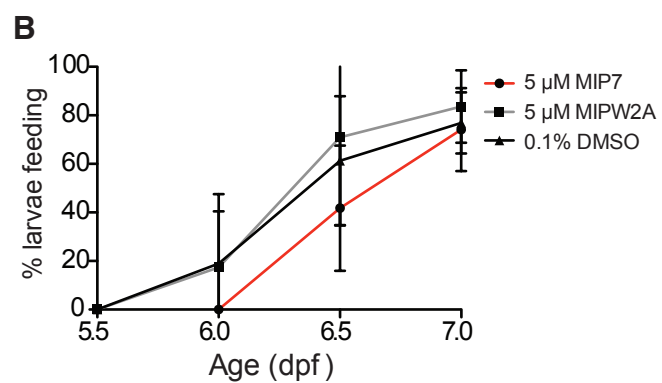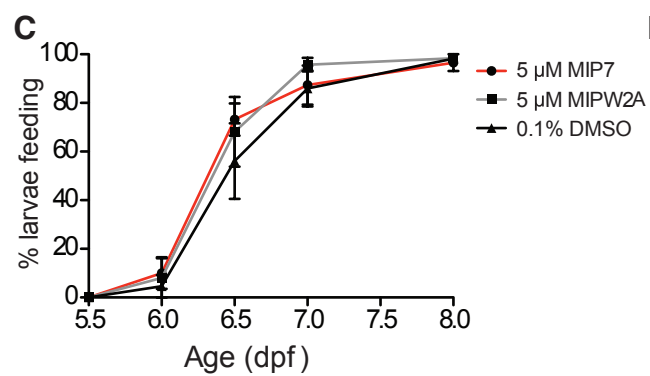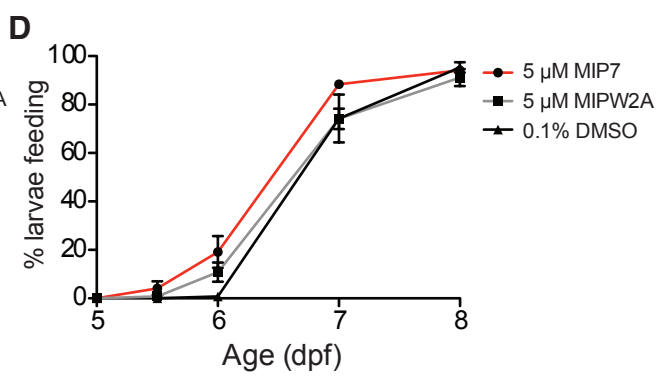

Supplement: Additional file 14 — Early treatment with MIP does not induce early onset feeding. Graphs of % larvae with food in gut over time after treatment with 5 μM MIP, 5 μM control MIPW2A or 0.1% DMSO from (A) 24 hpf, (B) 60 hpf, (C) 4 dpf, or (D) 5 dpf. Data are shown as mean +/- s.e.m., n = 3 x 30 larvae. p-value cut-offs based on unpaired t-tests indicated no significant difference in initiation of feeding between MIP-treated and control larvae. MIPW2A is a control non-functional MIP peptide in which the two conserved tryptophan sites are substituted with alanines. [file 12983_2014_93_MOESM14_ESM.pdf]

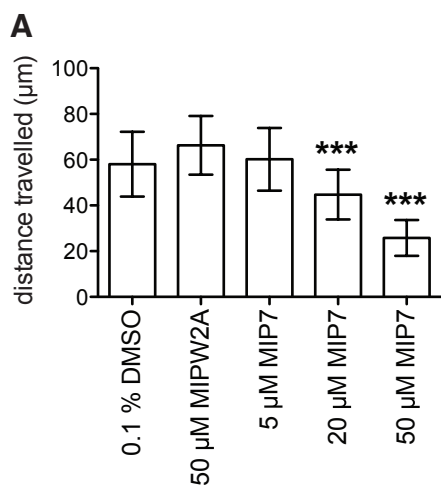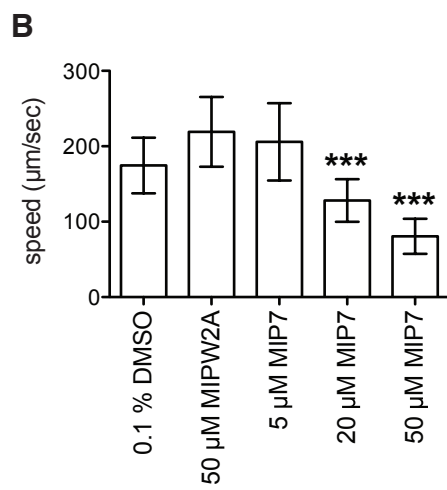

Supplement: Additional file 18 — MIP treatment decreases distance traveled and speed of 6 dpf Platynereis. (A) Distance travelled by MIP-treated versus control 6.5 dpf Platynereis. (B) Speed of MIP-treated versus control 6.5 dpf Platynereis. Data are shown as mean +/- 95% confidence interval, n = 60 larvae. p-value cut-offs based on unpaired t-test: *** <0.001; ** < 0.01; * <0.05. MIPW2A = control non-functional MIP peptide in which the two conserved tryptophan sites are substituted with alanines. [file 12983_2014_93_MOESM18_ESM.pdf]

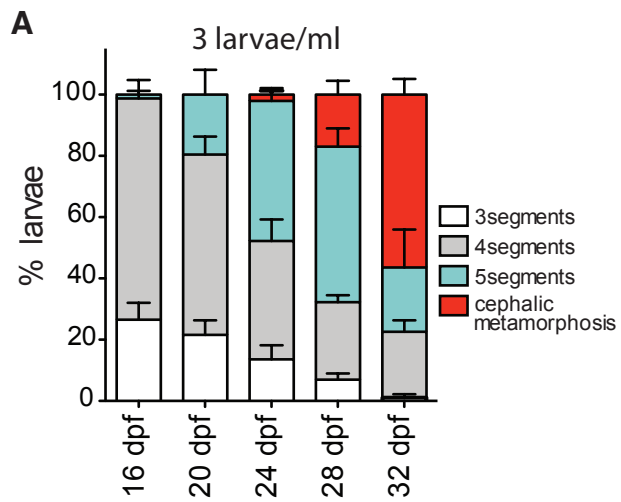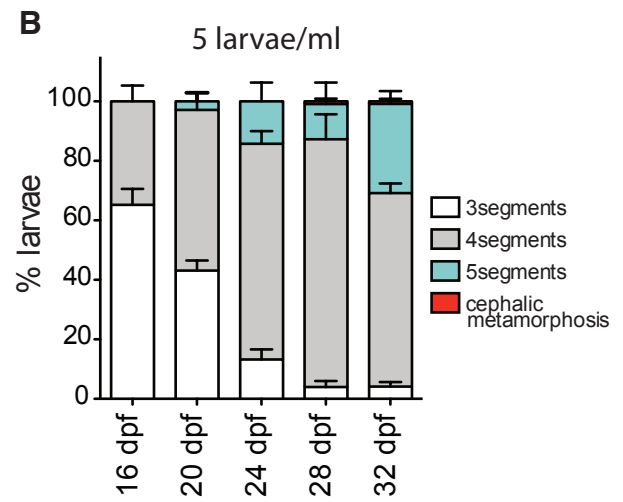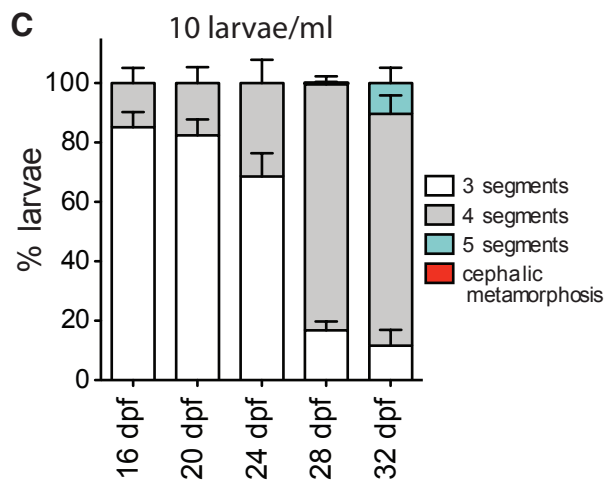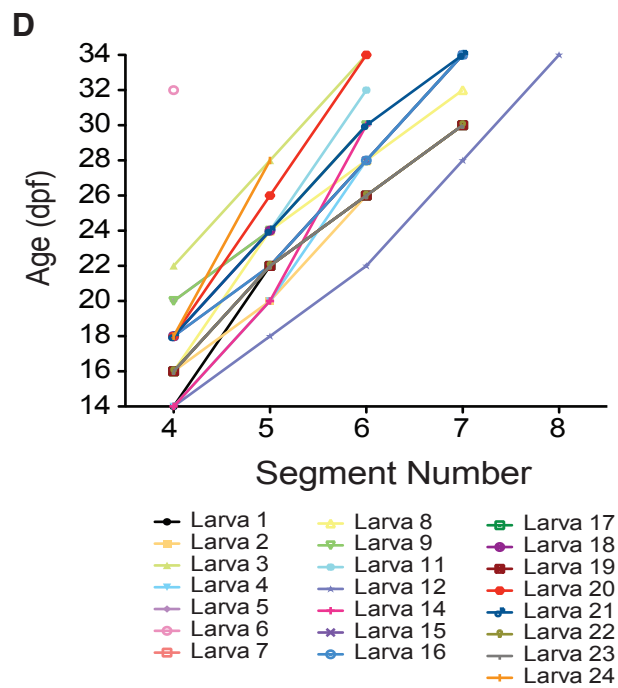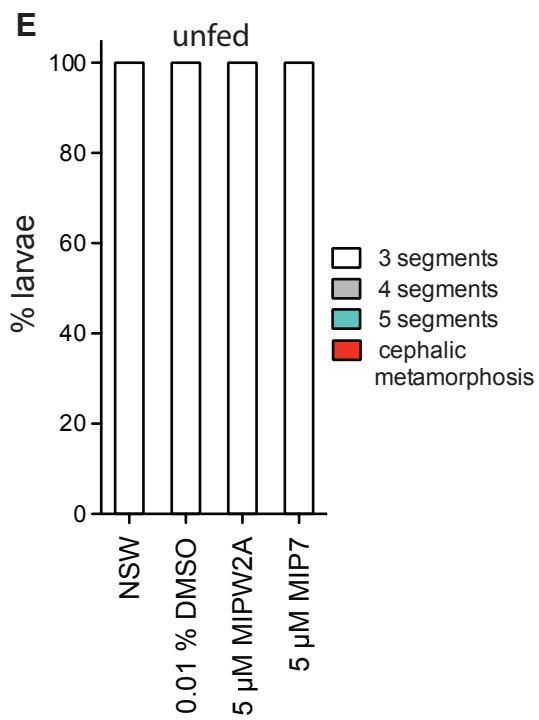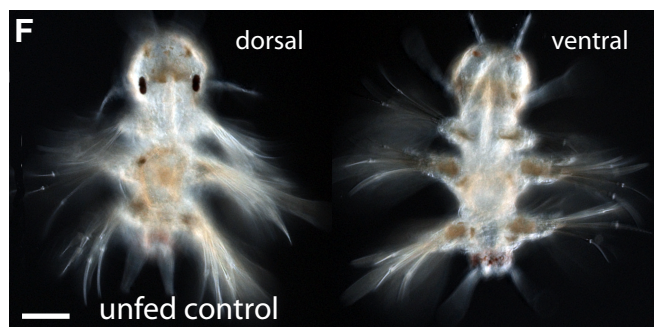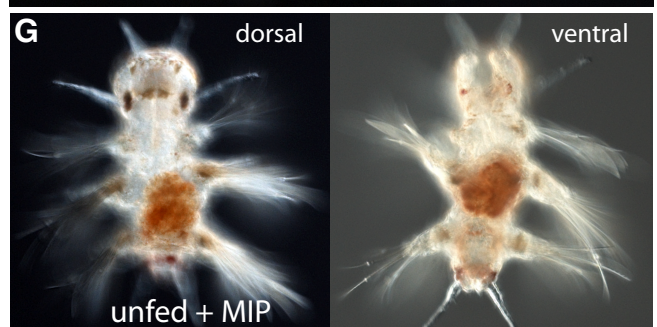

Supplement: Additional file 19 — Long-term growth of Platynereis. Addition of posterior segments in errant juveniles raised at a density of (A) 3 larvae/mL (30 larvae in 10mL NSW), n = 3 x 30, (B) 5 larvae/mL (50 larvae in 10 mL NSW), n = 3 x 50, or (C) 10 larvae/mL (100 larvae in 10 mL NSW), n = 3 x 100. (A-C) Data are shown as mean + s.e.m. All larvae were fed from 6 dpf onwards. ‘Cephalic metamorphosis’ encompasses all worms that have completed cephalic metamorphosis and have 5 or more chaetigerous segments. (D) Addition of posterior segments in errant juveniles raised individually. 24 larvae were raised individually in a 24-well tissue culture dish with 2 mL NSW per well. Note: Larva #13 died prior to 12 dpf. (E) No addition of posterior segments in unfed 5 μM MIP7-treated and control errant juveniles. Data shown are mean + s.e.m, n = 3 x 30 larvae. NSW, 0.22 μM filtered natural seawater. MIPW2A is a control non-functional MIP peptide in which the two conserved tryptophan sites are substituted with alanines. (F, G) Differential interference contrast (DIC) light micrographs of example unfed (F) control and (G) MIP-treated individuals at 24 dpf. Scale bar: 50 μm. [file 12983_2014_93_MOESM19_ESM.pdf]
