# Supplementary material for: Myoinhibitory peptide regulates feeding in the marine annelid Platynereis
Source: Front Zool. 2015 Jan 7;12:1. doi: 10.1186/s12983-014-0093-6 (PMC4307165; doi:10.1186/s12983-014-0093-6)

A

Amylase NJ-1000

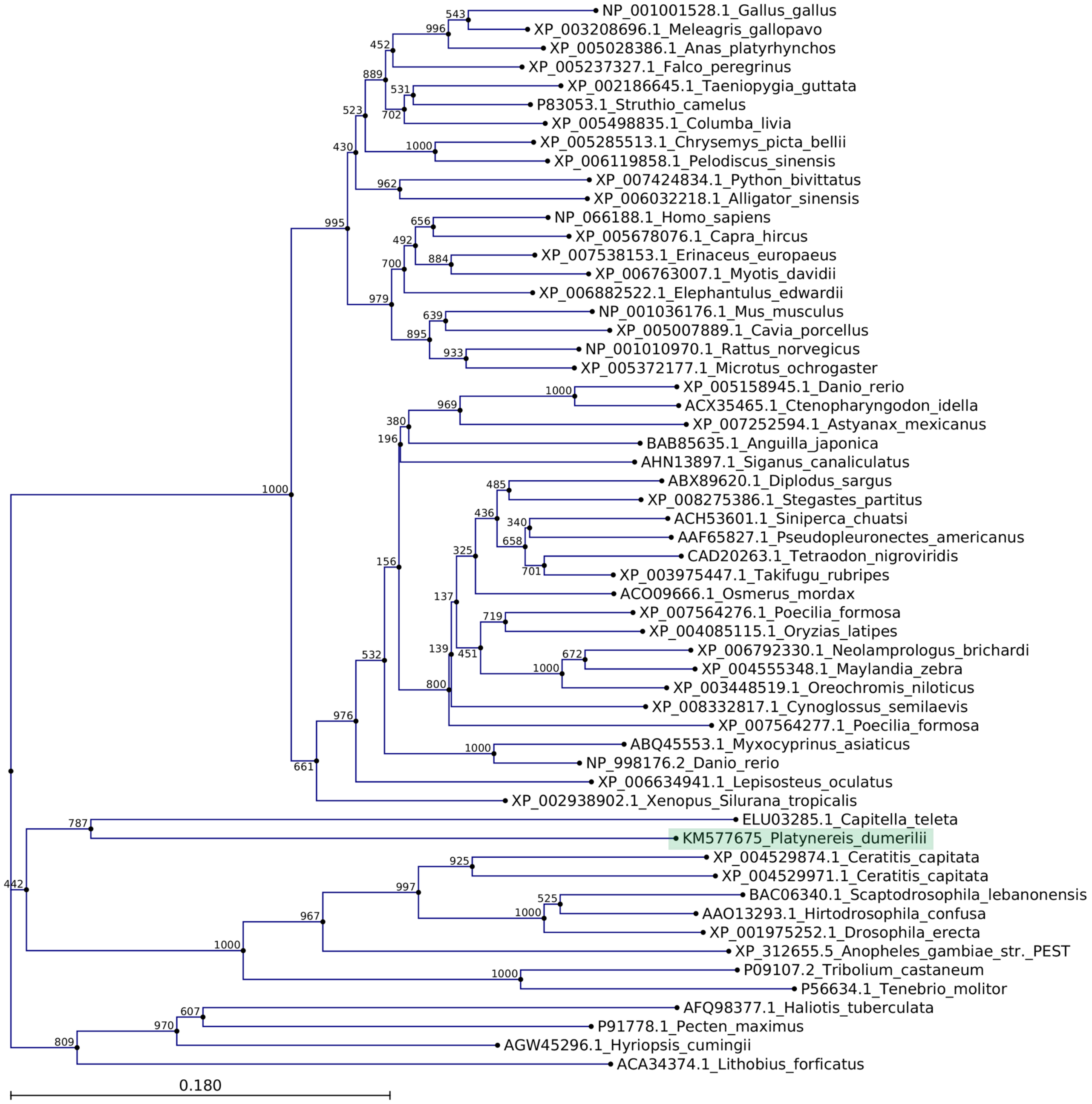

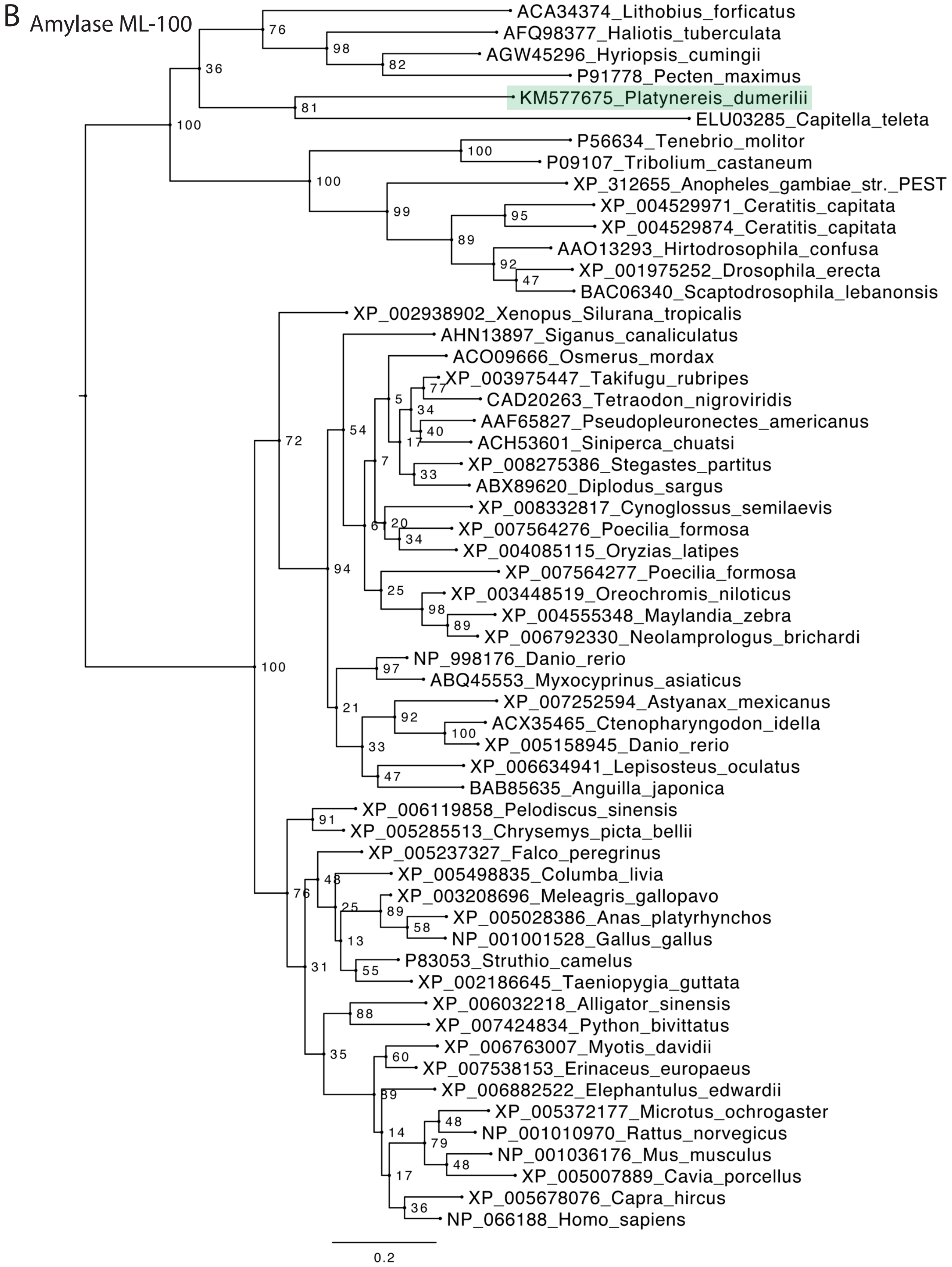
B
Amylase ML-100
ACA34374_Lithobius_forficatus
AFQ98377_Haliotis_tuberculata
AGW45296_Hyriopsis_cumingii
P91778_Pecten_maximus
KM577675_Platynereis_dumerilii
ELU03285_Capitella_teleta
P56634_Tenebrio_molitor
P09107_Tribolium_castaneum
XP_312655_Anopheles_gambiae_str._PEST
XP_004529971_Ceratitis_capitata
XP_004529874_Ceratitis_capitata
AAO13293_Hirtodrosophila_confusa
XP_001975252_Drosophila_erecta
BAC06340_Scaptodrosophila_lebanonsis
XP_002938902_Xenopus_Silurana_tropicalis
AHN13897_Siganus_canaliculatus
ACO09666_Osmerus_mordax
XP_003975447_Takifugu_rubripes
CAD20263_Tetraodon_nigroviridis
AAF65827_Pseudopleuronectes_americanus
ACH53601_Siniperca_chuatsi
XP_008275386_Stegastes_partitus
ABX89620_Diplodus_sargus
XP_008332817_Cynoglossus_semilaevis
XP_007564276_Poecilia_formosa
XP_004085115_Oryzias_latipes
XP_007564277_Poecilia_formosa
XP_003448519_Oreochromis_niloticus
XP_004555348_Maylandia_zebra
XP_006792330_Neolamprologus_brichardi
NP_998176_Danio_rerio
ABQ45553_Myxocyprinus_asiaticus
XP_007252594_Astyanax_mexicanus
ACX35465_Ctenopharyngodon_idella
XP_005158945_Danio_rerio
XP_006634941_Lepisosteus_oculatus
BAB85635_Anguilla_japonica
XP_006119858_Pelodiscus_sinensis
XP_005285513_Chrysemys_picta_bellii
XP_005237327_Falco_peregrinus
XP_005498835_Columba_livia
XP_003208696_Meleagris_gallopavo
XP_005028386_Anas_platyrhynchos
NP_001001528_Gallus_gallus
P83053_Struthio_camelus
XP_002186645_Taeniopygia_guttata
XP_006032218_Alligator_sinensis
XP_007424834_Python_bivittatus
XP_006763007_Myotis_davidii
XP_007538153_Erinaceus_europaeus
XP_006882522_Elephantulus_edwardii
XP_005372177_Microtus_ochrogaster
NP_001010970_Rattus_norvegicus
NP_001036176_Mus_musculus
XP_005007889_Cavia_porcellus
XP_005678076_Capra_hircus
NP_066188_Homo_sapiens
0.2

C

Enteropeptidase NJ-1000

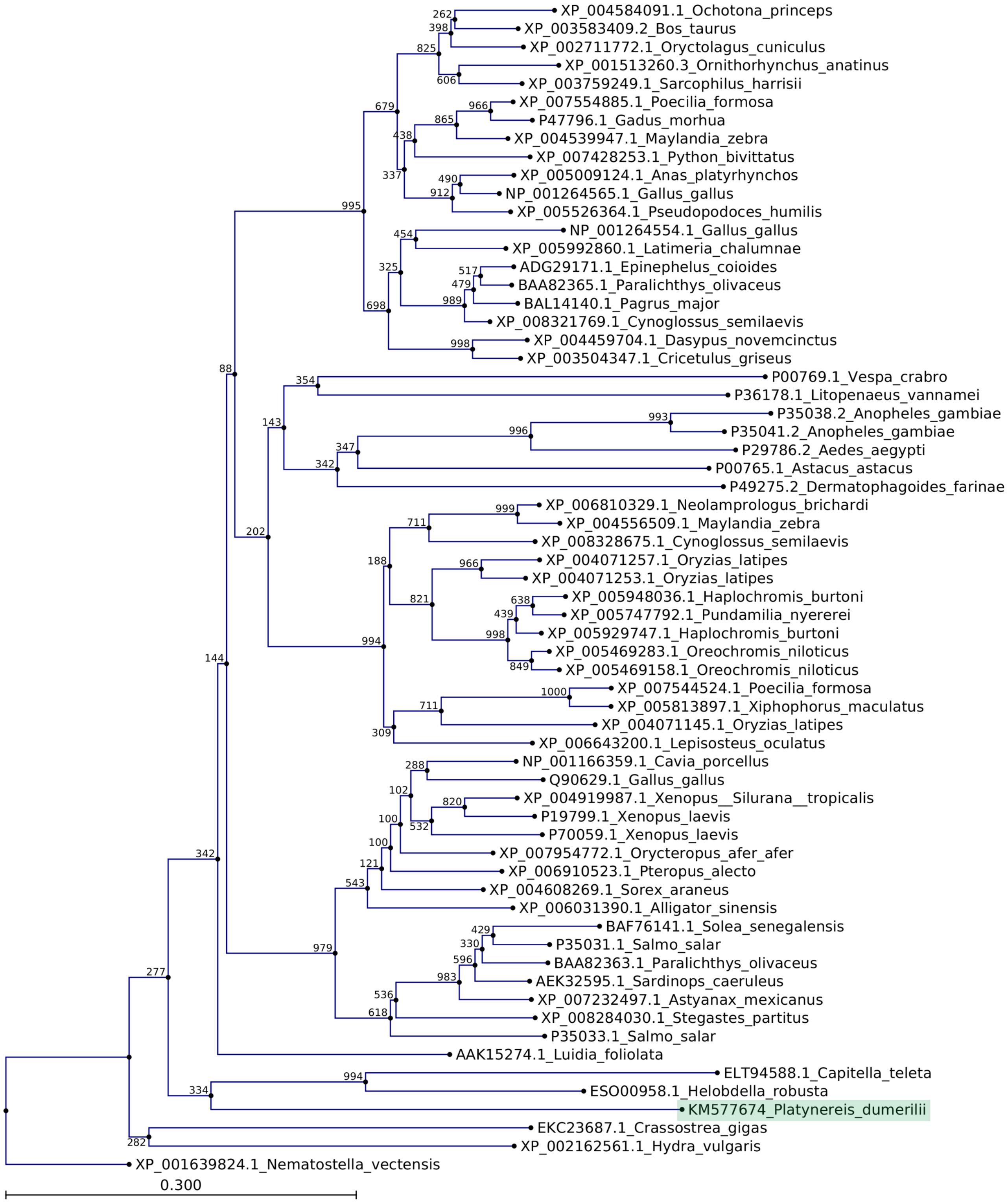

D

Enteropeptidase ML-100

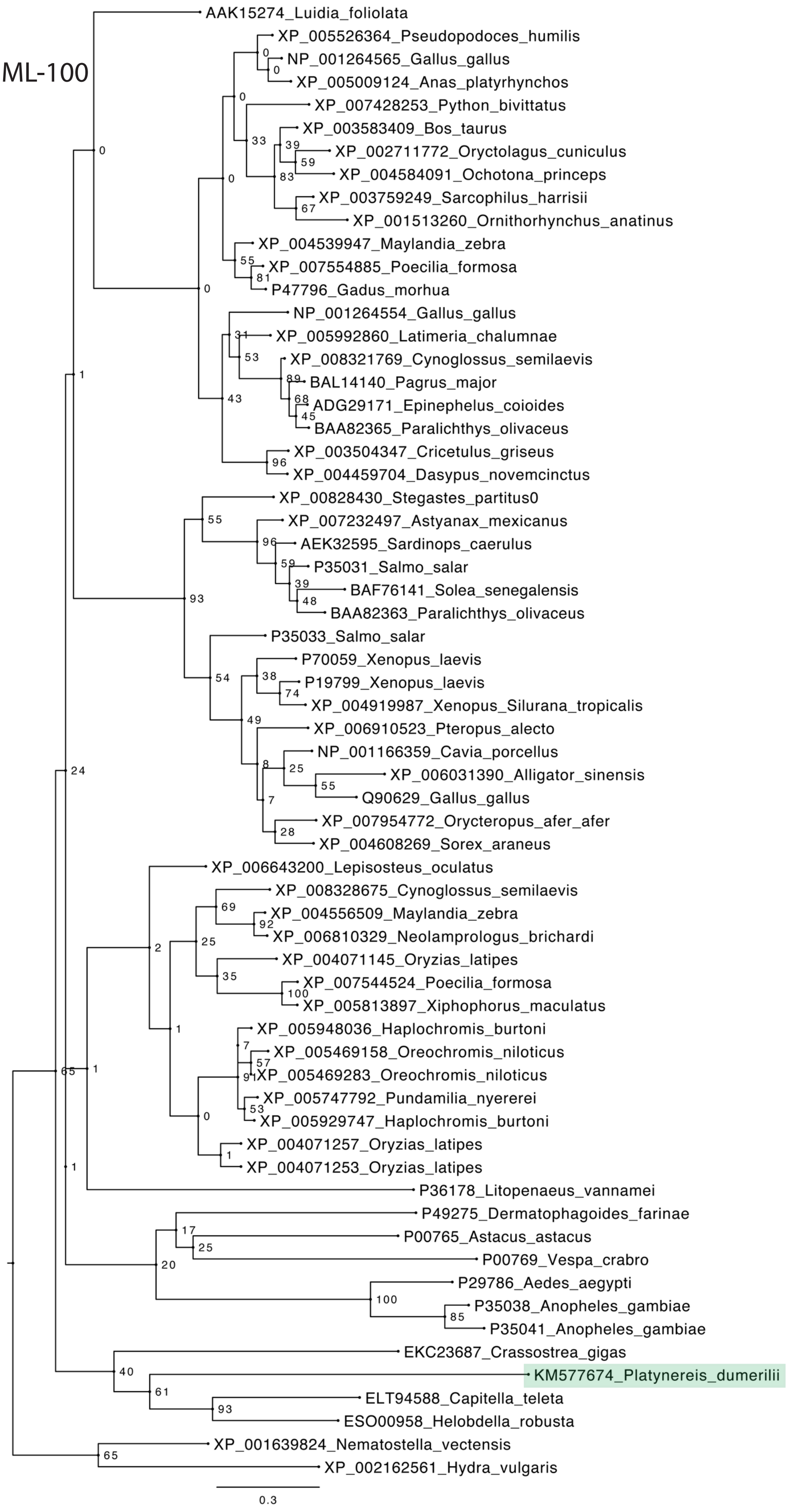

E Legumain NJ-1000

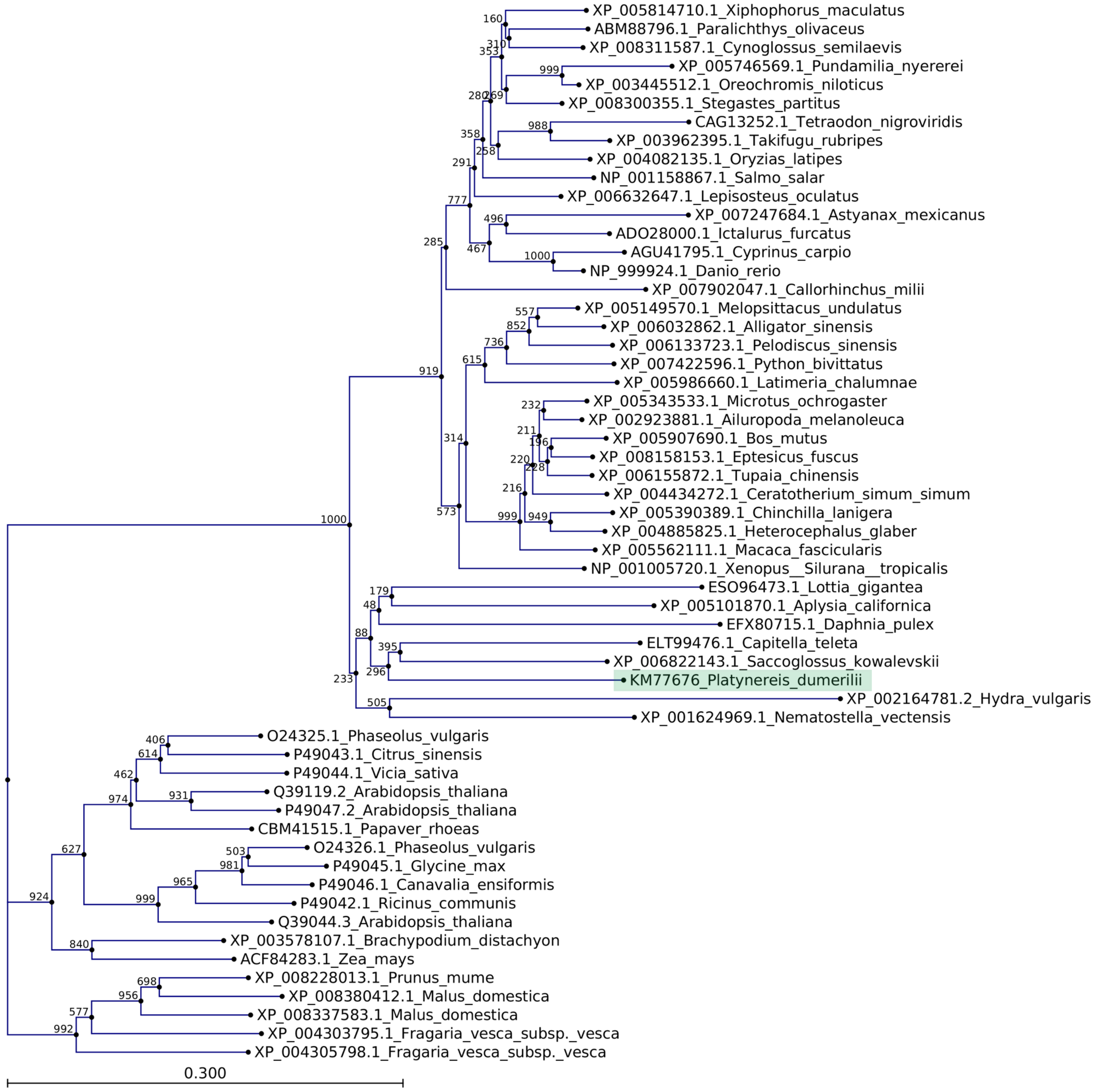

F

Legumain ML-100

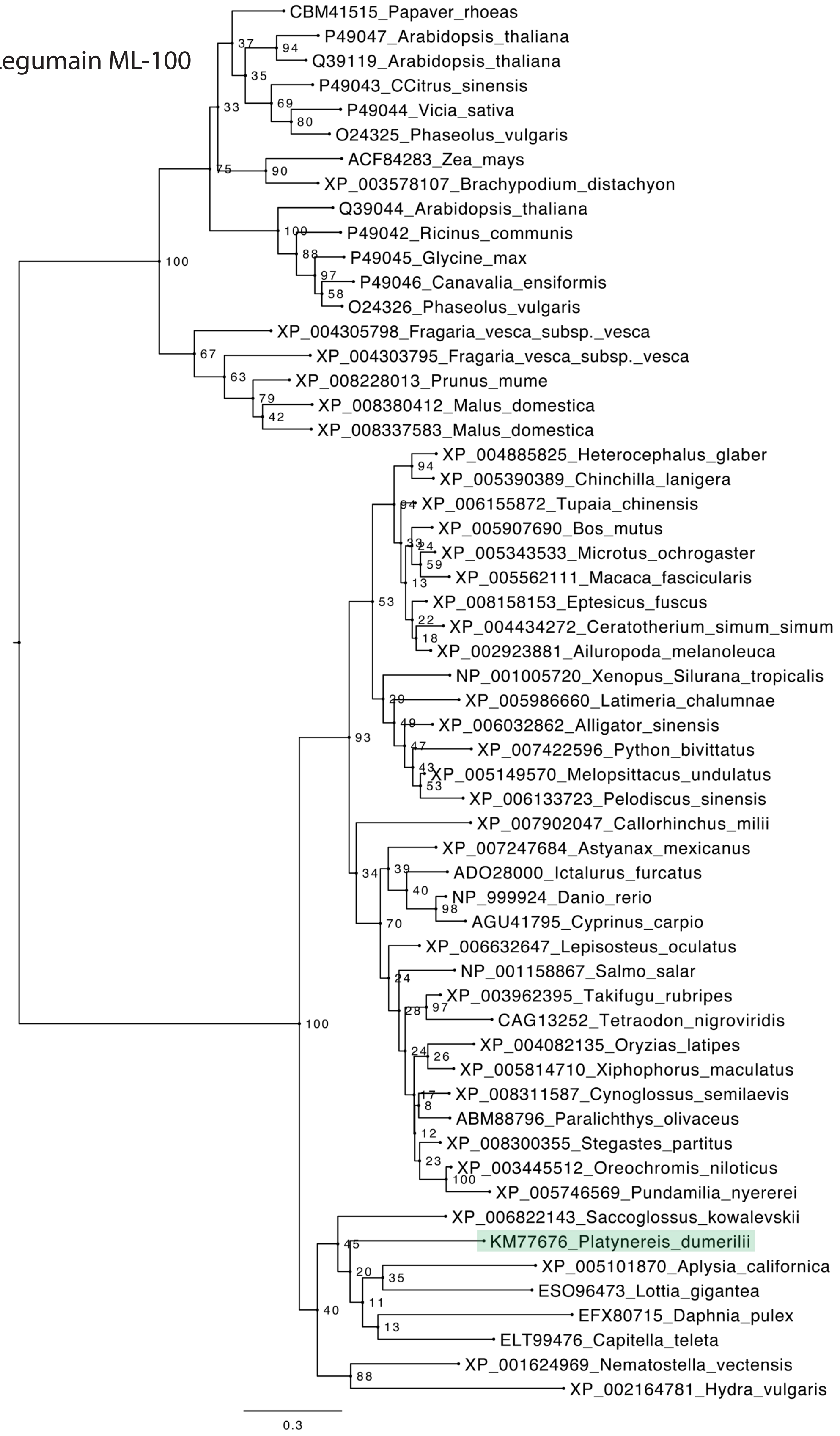

G

Subtilisin NJ-1000

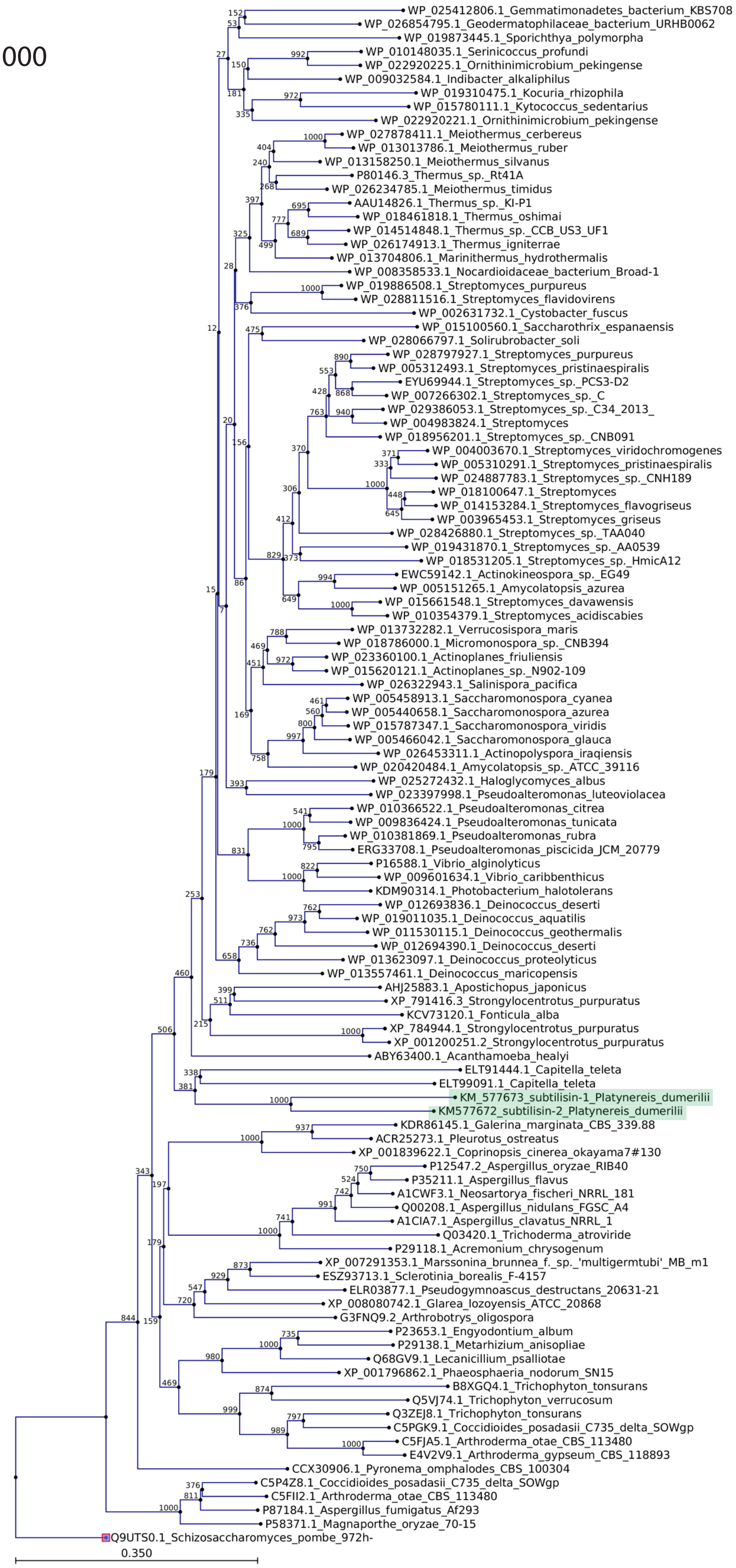

H

Subtilisin ML-100

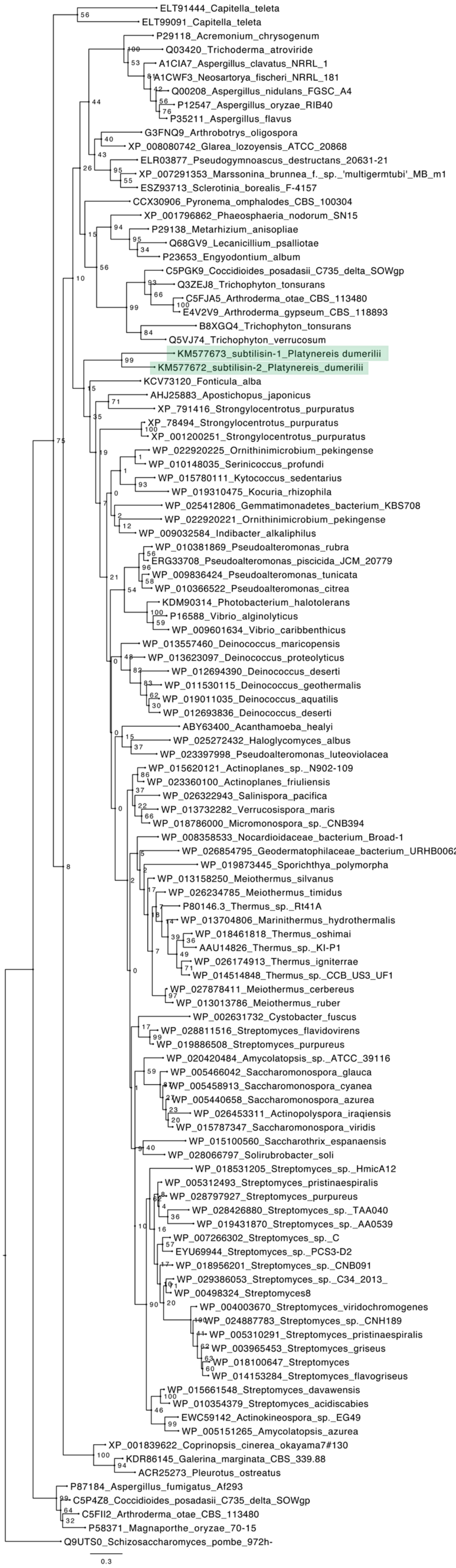

Supplement: Additional file 7 — Phylogenetic trees of Platynereis digestive system marker genes. Neighbour-joining trees with 1000 bootstrap repetitions (NJ-1000) and maximum likelihood trees with 100 bootstrap repetitions (ML-100) for digestive system markers alpha amylase (A, B), enteropeptidase (B, C), legumain protease precursor (D, E), and subtilisin-1 and subtilisin-2 (F, G). The positions of the Platynereis candidate genes are highlighted by green boxes. Bootstrap values are indicated at branch nodes. Platynereis alpha amylase and legumain protease precursor cluster with their counterparts in a fellow polychaete, Capitella teleta, within invertebrate-specific clades. The orthology of these genes was confirmed by reciprocal BLAST to the Homo sapiens peptidome. Platynereis enteropeptidase clusters in a weakly-supported group of annelid enteropeptidases, however the identity of this gene is confirmed by the presence of conserved MAM and trypsin domains (Additional file 6) [49]. Platynereis subtilisin-1 and subtilisin-2 are intermingled with several bacterial, fungal, annelid and echinoderm sequences in a poorly resolved tree, suggesting a possible horizontal gene transfer event. Subtilisin-1 and –2 contain conserved peptidase domains (Additional file 6), including the presence of a catalytic triad, suggesting that they maintain enzymatic function in Platynereis. [file 12983_2014_93_MOESM7_ESM.pdf]
