# Supplementary material for: Myoinhibitory peptide regulates feeding in the marine annelid Platynereis
Source: Front Zool. 2015 Jan 7;12:1. doi: 10.1186/s12983-014-0093-6 (PMC4307165; doi:10.1186/s12983-014-0093-6)
Supplement: Additional file 11 — Target sites of Platynereis MIP start morpholinos. The sequence of the 5′ region of the Platynereis MIP precursor gene is shown. Binding sites of MIP start morpholinos (MOs) 1 and 2 are outlined in red. The start codon for the MIP precursor peptide is underlined and in bold. The 5′ predicted peptide sequence is indicated in grey. MIP start MO1 binds to the start codon region of the gene, while MIP start MO2 binds in the upstream 5′-UTR region. [file 12983_2014_93_MOESM11_ESM.pdf]

Platynereis MIP precursor

5′- CTGCAGCGCCCACTGCATAAGTGCGTGGGACAGTATTTCAACAGACAGCCACTATAA

MIP start MO2

AGATAAGAGGGAGAGAAGGAACTAG

ATATATCGAAGAAAGTTTAATCCTGTTAGTTGGAGATAAGAGGGAGAGAAGGAACTAG

TGTTTGGAACAACTCTTTATAGGAAACAAGGAACACACAAAGAAGCAACAAGGAA

MIP start MO1

AGTCCA**ATG**GATCGCGTCA

GAAAAGGAAAGAATATTAATACAACCAAGGGCCCTAGCAGTCCA**ATG**GATCGCGTCA

M D R V

CTATCA

CTATCACCTGCTTCTCCCTCTGTCTGGCGTCA… -3′

T I T C F S L C L A S
